# Anticancer and antimicrobial activity of biosynthesized Red Sea marine algal silver nanoparticles

**DOI:** 10.1038/s41598-022-06412-3

**Published:** 2022-02-14

**Authors:** Rabaa Algotiml, Ali Gab-Alla, Roshdi Seoudi, Hussein H. Abulreesh, Mahmoud Zaki El-Readi, Khaled Elbanna

**Affiliations:** 1 Department of Biology, Faculty of Applied Science, Umm Al-Qura University, Makkah, Saudi Arabia; 2 Research Laboratories Unit, Faculty of Applied Science, Umm Al-Qura University, Makkah, Saudi Arabia; 3 Department of Marine Science, Faculty of Science, Suez Canal University, Ismailia, Egypt; 4 Spectroscopy Department, Physics Division, National Research Center, Dokki, Cairo, 12622 Egypt; 5 Department of Clinical Biochemistry, Faculty of Medicine, Umm Al-Qura University, Makkah, 21955 Saudi Arabia; 6 Department of Biochemistry, Faculty of Pharmacy, Al-Azhar University, Assiut, 71524 Egypt; 7 Department of Agricultural Microbiology, Faculty of Agriculture, Fayoum University, Fayoum, 63514 Egypt

**Keywords:** Biological techniques, Chemical biology, Microbiology

## Abstract

Biosynthesis of silver nanoparticles (AgNPs) is emerging as a simple and eco-friendly alternative to conventional chemical synthesis methods. The role of AgNPs is expanding as antimicrobial and anticancer agents, sensors, nanoelectronic devices, and imaging contrast agents. In this study, biogenic AgNPs were synthesized using extracts of different marine algae species, including *Ulva rigida* (green alga), *Cystoseira myrica* (brown alga), and *Gracilaria foliifera* (red alga), as reducing and capping agents. The Physiochemical properties, cytotoxicity, anticancer and antimicrobial activities of the biosynthesized AgNPs were assessed. Surface plasmonic bands of the biosynthesized AgNPs capped with *U. rigida*, *C. myrica*, and *G. foliifera* extracts were visually observed to determine a colour change, and their peaks were observed at 424 nm, 409 nm, and 415 nm, respectively, by UV–Vis spectroscopy; transmission electron microscopy (TEM) indicated an almost spherical shape of AgNPs with nanoscale sizes of 12 nm, 17 nm, and 24 nm, respectively. Fourier transform-infrared (FTIR) spectroscopy analysis suggested that different molecules attached to AgNPs through OH, C=O, and amide groups. The major constituents of the aqueous algal extracts included, terpenoids, polyphenols, sulfonates, polysaccharides, fatty acids, chlorophylls, amide proteins, flavonoids, carotenoids, aliphatic fluoro compounds, volatile compounds, alkalines, pyruvic acid and agar groups. The cytotoxicity and anticancer activities of the biosynthesized AgNPs were assessed using *Artemia salina* nauplii, normal skin cell lines (HFb-4), and breast cancer cell lines (MCF-7 cell line). The lethality was found to be directly proportional to the AgNP concentration. The IC_50_ values of *C. myrica* and G*. foliifera* AgNPs against *A. saline* nauplii were 5 and 10 μg ml^−1^ after 4 h and 16 h, respectively, whereas *U. rigida* AgNPs did not exhibit cytotoxic effects. Anticancer activity of the biosynthesized AgNPs was dose dependent. The IC_50_ values of the biosynthesized AgNPs were 13, 13, and 43 µg ml^−1^ for *U. rigida, C. myrica,* and *G. foliifera*, respectively. *U. rigida* AgNPs particularly exhibited potent anticancer activity (92.62%) against a human breast adenocarcinoma cell line (MCF-7) with high selectivity compared the normal cells (IC_50_ = 13 µg/ml, SI = 3.2), followed by *C. myrica* AgNPs (IC_50_ = 13 µg/ml, SI = 3.07). Furthermore, the biosynthesized AgNPs exhibited strong antifungal activity against dermatophyte pathogenic moulds and mild antibacterial activity against the food borne pathogen bacteria. The highest antimicrobial activity was recorded for the *U. rigida* AgNPs, followed by those capped with *C. myrica* and *G. foliifera* extracts, respectively. AgNPs capped with the *U. rigida* extract exhibited the highest antimicrobial activity against *Trichophyton mantigrophytes* (40 mm), followed by *Trichosporon cataneum* (30 mm) and *E. coli* (19 mm), with minimal lethal concentration of 32 and 64 μg ml^−1^ respectively. The study finally revealed that extracts of marine algal species, particularly *U. rigida* extracts, could be effectively used as reducing agents for the green synthesis of AgNPs. These AgNPs are considered efficient alternative antidermatophytes for skin infections and anticancer agents against the MCF-7 cell line.

## Introduction

Nanoscale particles 1–100 nm in size are known as nanoparticles (NPs). NPs exhibit unique physical, chemical, and biological properties compared to bulk materials and possess crystal structures, higher surface-to-volume ratios, and controlled targeting and release properties. The fields of drug delivery, cosmetology, pharmacy, biotechnology, tissue engineering, chemistry, agriculture, and diagnostics are rapidly expanding the application of NPs^1,2^. Silver NPs (AgNPs) have gained increasing attention for multiple applications as antimicrobial, antioxidants, anticancer, antidiabetic, imaging contrast, sensors agents, in nanoelectronic devices, in filters, in water purification, in environmental pollution control, as well as enhancing vaccine immunogenicity, wound and bone healing^3–9^.

Emerging multidrug resistance (MDR) in gram-positive and gram-negative bacteria has promoted the development of novel antibacterial agents. The small size and large surface area of AgNPs causes them to exhibit broad-spectrum, strong antimicrobial activities against various microorganisms. Low concentrations of AgNPs can efficiently kill bacterial and viral pathogens, including *Escherichia coli*, *Staphylococcus aureus, Klebsiella pneumoniae*, *Candida albicans*, *Aspergillus nige*r, human immunodeficiency virus (HIV), and hepatitis B virus (HBV)^10,11^. AgNPs also play an efficient role during in vitro and in vivo treatments of lung cancer, cervical cancer, hepatocellular carcinoma, breast cancer, nasopharyngeal carcinoma, prostate carcinoma, colorectal adenocarcinoma, and glioblastoma^4,12^. Recently, AgNPs have been used in anticancer therapies of the Hep-2 cell line, HT-29 cell lines^13^ , Vero cell line^14^, and breast cancer line MCF-7^15^.

The activity of reducing reagents on silver ions leads to the production of AgNPs. Different physical, chemical, and mechanical methods have been developed to manufacture NPs^3^. However, in addition to high cost and non-eco-friendly impacts, other hazards, such as cytotoxicity, genotoxicity, and carcinogenicity, are also associated with these practices^16^. Therefore, the synthesis of eco-friendly NPs is urgently needed to replace toxic chemicals in various fields. Biosynthesized AgNPs are a cost-effective class of eco-friendly biocompatible agents that possess the potential for biomedical and pharmaceutical applications^5–8^. Microorganisms such as wild mushroom, algae, and bacteria, as well as plant extracts, contain enzymes, alkaloids, terpenoids, and phenolic compounds that can be used as stabilizers and capping agents during the biological synthesis of NPs ^6,17^. Currently, marine resources are being increasingly explored for antimicrobial and anticancer drug candidates due to their relatively mild side effects^18^. Marine algae, including Chlorophyta (green), Phaeophyta (brown), and Rhodophyta (red), are considered highly potent renewable living marine resources that have received increasing interest for the biosynthesis of NPs. The organic compounds in algae, including polysaccharides, proteins, carbohydrates, vitamins, pigments, enzymes, and secondary metabolites, give further potential to their role in the biosynthesis of AgNPs by acting as natural reducing agents^19–21^. These active biomolecules can be used to prepare controlled AgNPs of different shapes and sizes, such as spheres, wires, rod triangles, cubes, hexagons, and pentagons. Amino acids, proteins, and sulfated polysaccharides in algal extracts with variable properties can also act as capping agents or stabilizers in the biosynthesis of AgNPs^22^. The abundant organic content, rapid growth, and high metal accumulation abilities of different algae make them ideal candidates for the biosynthesis of AgNPs^22^. The present work aimed to (I) synthesize AgNPs using different algal species collected from the Red Sea coast of Saudi Arabia, (II) characterize the physicochemical properties of synthesized NPs, (III) assess cytotoxicity using *Artemia salina* and normal a skin cell line (HFb-4) and evaluate their antifungal and antibacterial activities against foodborne pathogens and dermatophytic fungi, and (IV) assess their anticancer potential against the human breast adenocarcinoma cell line MCF-7.

## Results

**Figure 1:**
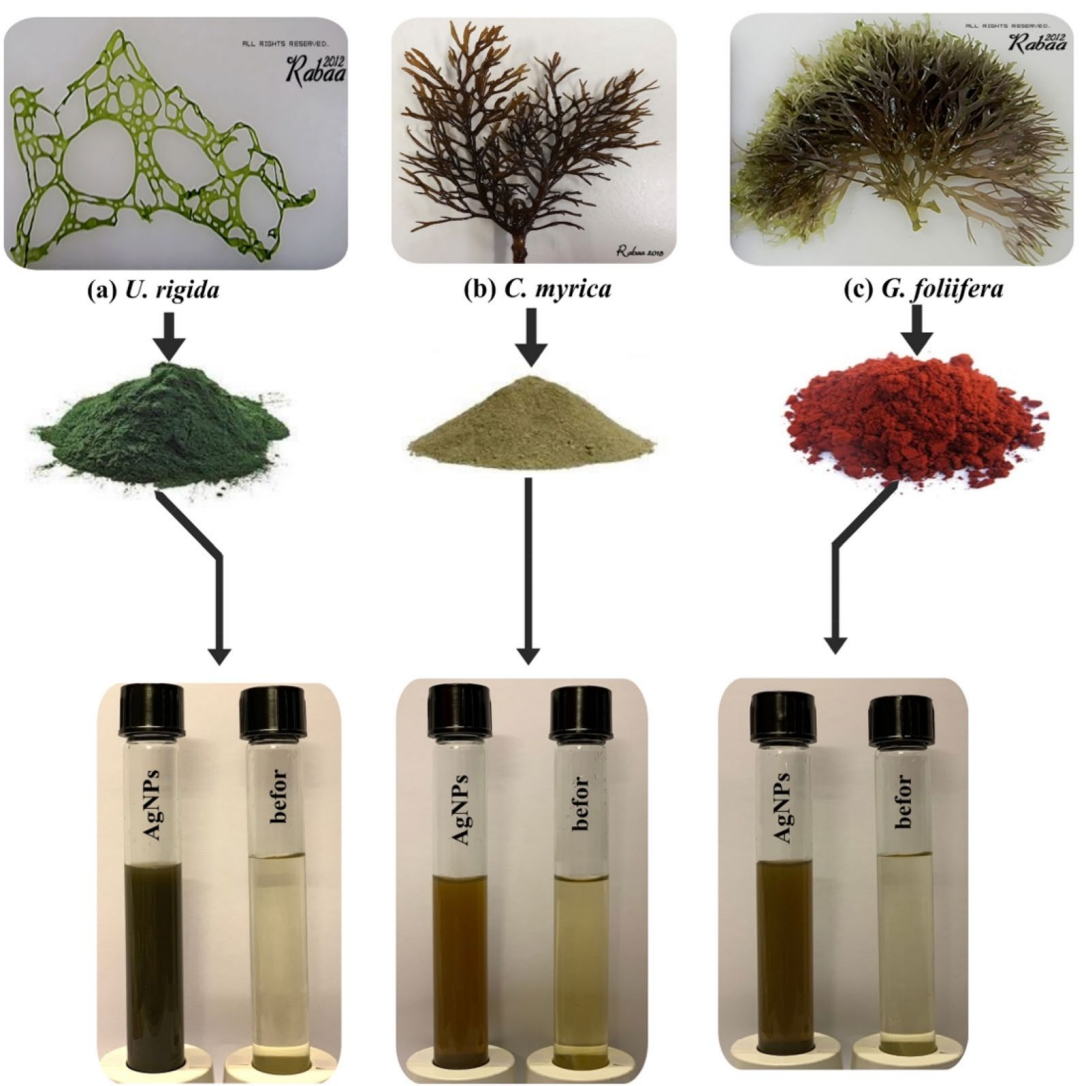
Biosynthesis of AgNPs using different marine algal extracts, (**a**) *U. rigida*, *C. myrica,* and *G. foliifera*, as reducing and capping agents. The reduction of AgNO_3_ by the algal extracts was visually evident from the colour change (brownish-yellow) of the reaction mixture after 48 h.

**Figure 2:**
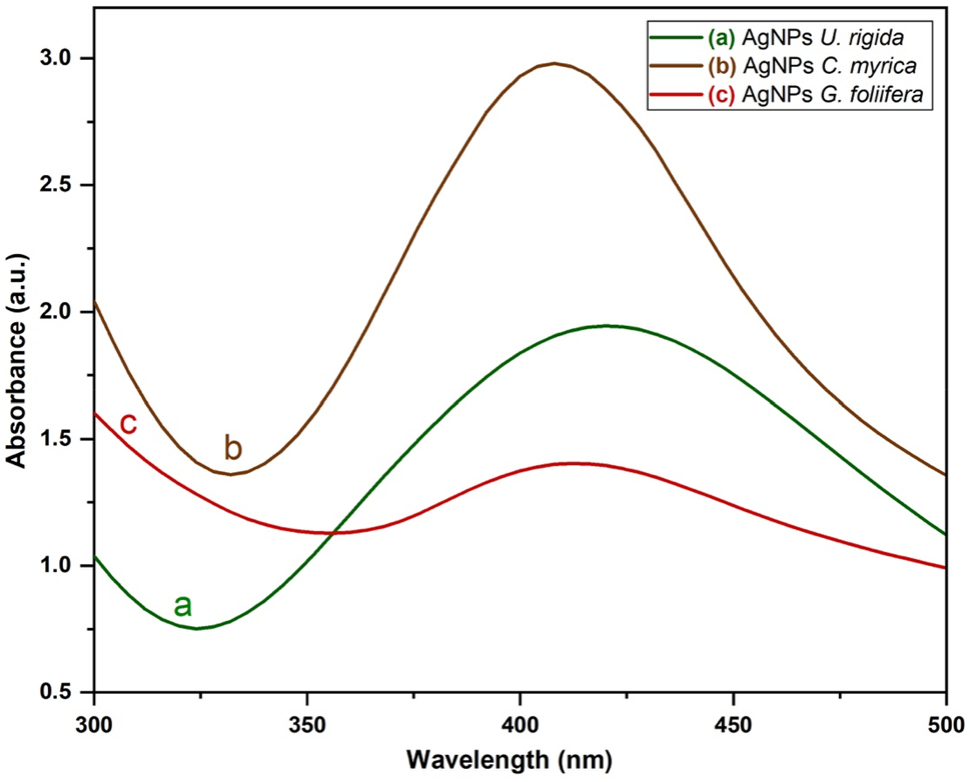
UV–vis spectra of the biosynthesized AgNPs capped by different marine algal extracts: (**a**) *U. rigida,* (**b**) *C. myrica* and (**c**) *G. foliifera***.**

The physiochemical characterization of biosynthesized AgNPs capped by different algal species was carried out via UV–vis spectroscopy, transmission electron microscopy (TEM), and Fourier-transform infrared (FTIR) spectroscopy. The reduction of AgNO_3_ by *U. rigida*, *C. myrica,* and *G. foliifera* was visually evident from the colour change (brownish-yellow) of the reaction mixture after 48 h. The intensity of the brown colour was directly correlated to the incubation period (Fig. 1). UV–Vis spectra of AgNPs formed by the extracts of different algal species are shown in Fig. 2. Absorption peaks of AgNPs capped by *U. rigida*, *C. myrica,* and *G. foliifera* appeared at 424 nm, 409 nm, and 415 nm, respectively.

**Figure 3:**
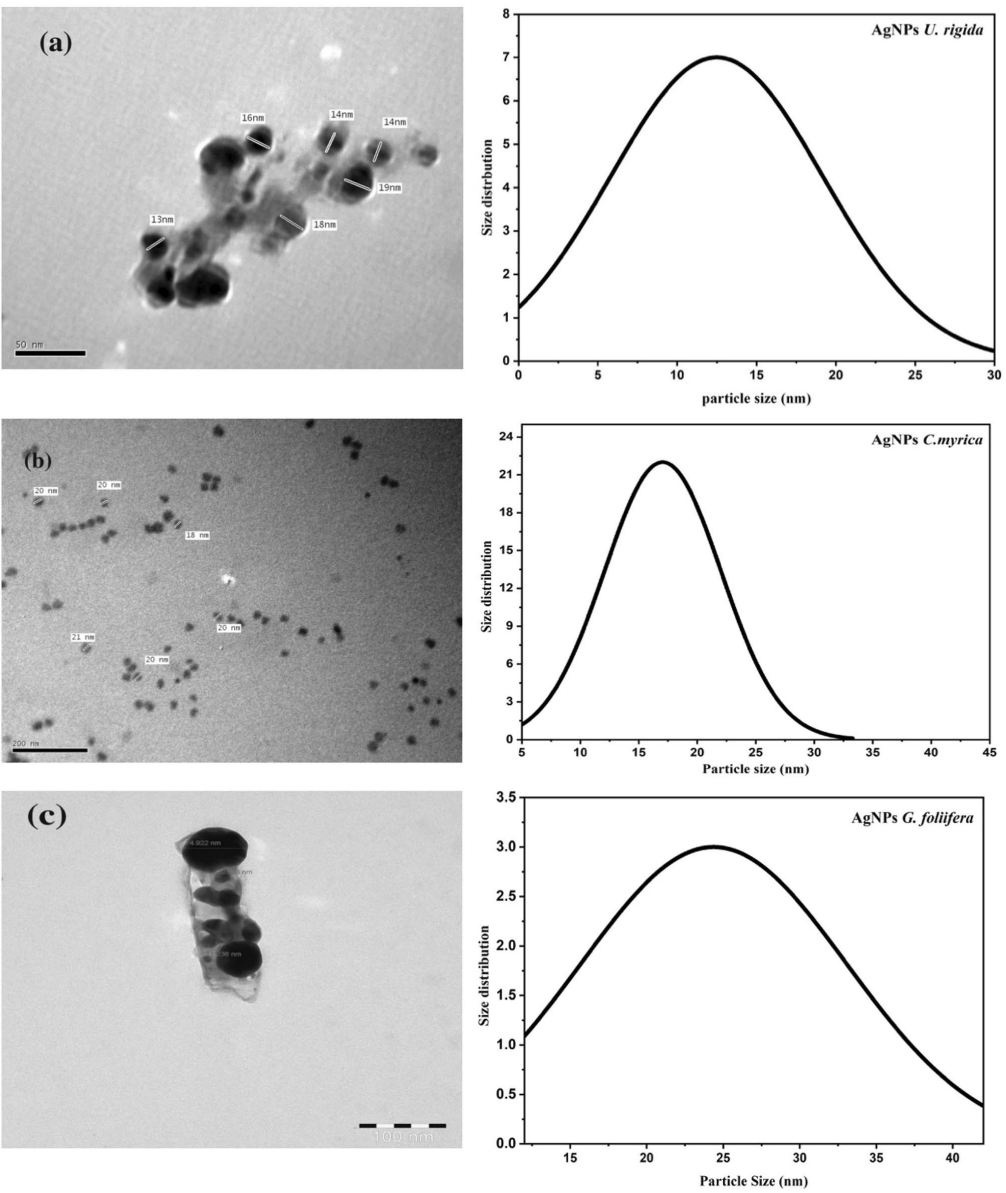
TEM images of the biosynthesized AgNPs capped by different marine algal extracts: (**a**) *U. rigida,* (**b**) *C. myrica* and (**c**) *G. foliifera***.**

Figure 3 presents TEM images of AgNPs stabilized by *U. rigida, C. myrica*, and *G. foliifera.* AgNPs capped by* U. rigida* were mostly spherical or pentagonal (Fig. 3a), were well distributed without aggregation in solution, and had an average size of 12.6 nm, as shown in the Gaussian distribution (Fig. 3a). TEM images of AgNPs stabilized by *C. myrica* are shown as spherical, grey and dark points (Fig. 3b) with an average particle size of 17 nm. TEM images showed that AgNPs stabilized by *G. foliifera* were also mostly spherical with some aggregation and triangular in shape and had an average size of 24.5 nm (Fig. 3c).

**Figure 4:**
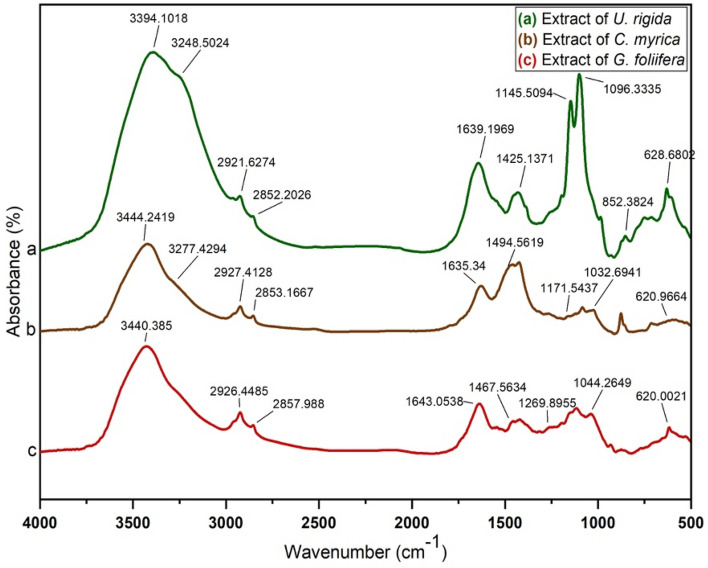
FTIR spectra of the extracts from different marine algal species: (**a**) *U. rigida,* (**b**) *C. myrica* and (**c**) *G. foliifera***.**

**Figure 5:**
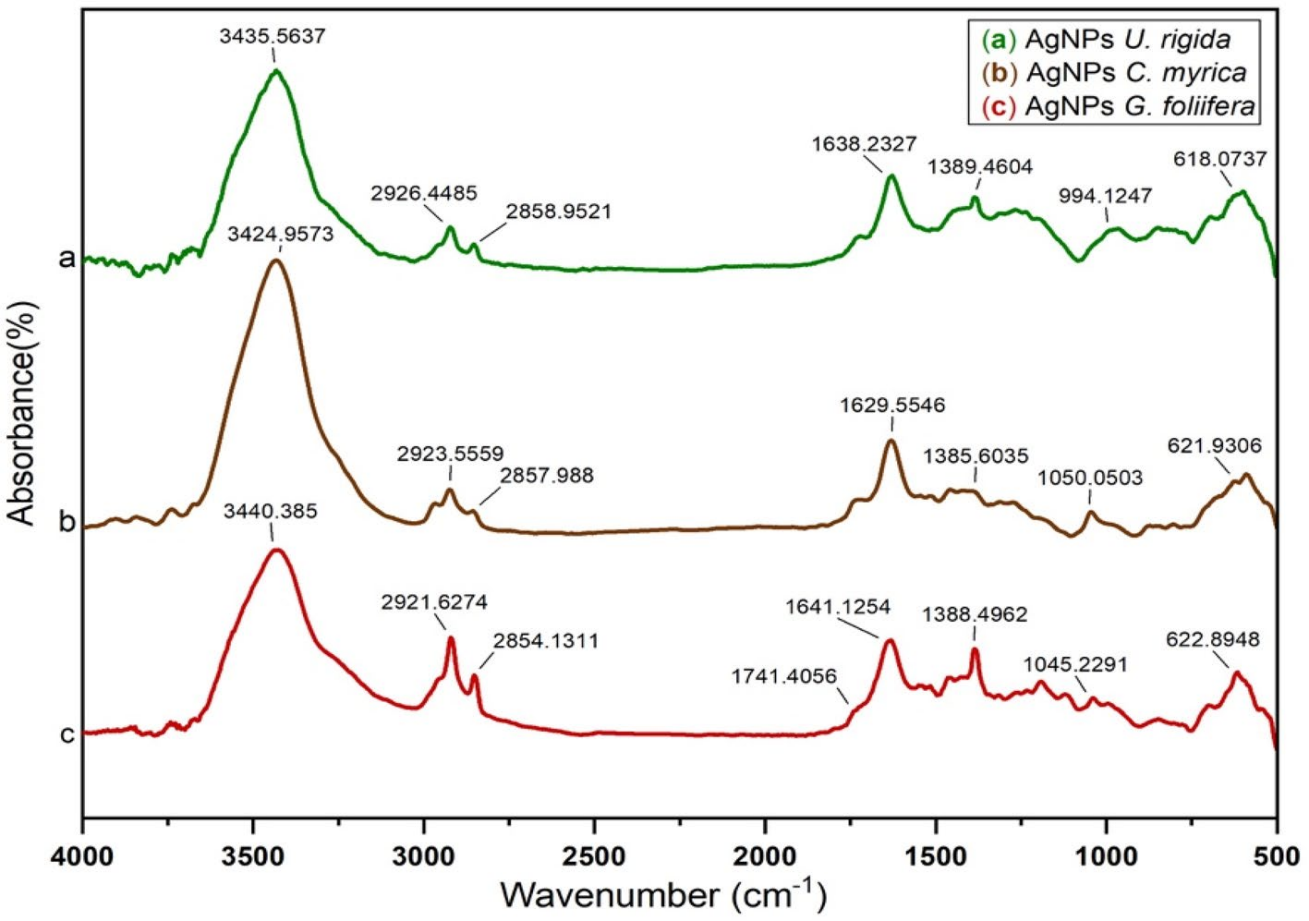
FTIR spectra of the biosynthesized AgNPs capped by different marine algal extracts: (**a**) *U. rigida,* (**b**) *C. myrica* and (**c**) *G. foliifera***.**

FTIR spectra of *U. rigida*, *C. myrica,* and* G. foliifera* extracts were measured in the wavenumber range of 4000–500 cm^−1^. The *U. rigida* spectrum (Fig. 4a) exhibited a prominent peak at 3394 cm^−1^, indicating the presence of hydroxide group O–H stretching vibrations. The band at 3248 cm^−1^ was interpreted as the N–H stretching vibration of proteins and terpenoids in *U. rigida.* The two peaks at 2919 cm^−1^ and 2852 cm^−1^ were revealed as asymmetric and symmetric stretching vibrations of CH_3_, whereas the peak at 1425 cm^−1^ presented the C=C vibration. The band at 1232 cm^−1^ represented the reconciling of the N–C group stretching vibrations. The peak at 1096 cm^−1^ was due to the rocking bending vibrations of C-F, whereas the peak at 852 cm^−1^ corresponded to C–C. The band at 628 cm^−1^ was attributed to the bending vibrations of C-H. Figure 4b shows the spectrum of *C. myrica*. The absorbance band observed at approximately 3444 cm^−1^ was recognized as hydroxyl group O–H stretching, whereas the band at approximately 3277 cm^−1^ was related to N–H stretching vibrations. The two peaks at 2927 cm^−1^ and 2853 cm^−1^ depicted the asymmetric and symmetric stretching vibrations of CH_3_, respectively. The absorption band at 1750 cm^−1^ explained the stretching vibration of C=C, whereas the band at 1635 cm^−1^ was interpreted as the C=O stretching vibration of *C. myrica*. The peak at 1494 cm^−1^ was assigned to the C-O- group vibrations, and the peak at 1032 cm^−1^ was appointed to the rocking-bending vibrations of CH_2_. The band at approximately 809 cm^−1^ presented the characteristic stretching vibrations of the O-S–O group. The infrared spectrum of *G. foliifera* (Fig. 4c) showed an N–H stretching vibration band at 3269 cm^−1^. The two bands observed at approximately 2926 cm^−1^ and 2858 cm^−1^ were assigned to asymmetric and symmetric stretching vibrations of CH_2_. The band at 1745 cm^−1^ explained the C=C group stretching vibrations. The peak seen at 1643 cm^−1^ was interpreted as the C=O stretching vibration, and the peak at 1467 cm^−1^ corresponded to the S=O group vibrations. The band at 1317 cm^−1^ illustrated the stretching vibration of C-N-, whereas the band at 1124 cm^−1^ was related to the rocking bending vibrations of the *G. foliifera* S–O structure*.* The band at approximately 1044 cm^−1^ exhibited the stretching vibration of CH_3_, and the band at approximately 620 cm^−1^ presented the stretching vibrations of the C–O–C group in the *G. foliifera* structure. Figure 4 indicates that terpenoids, polyphenols, carotenoids, fatty acids, carbohydrates, and lipids were the dominant biomolecules in the *U. rigida* extract, whereas polyphenols, sulfonated polysaccharides (fucoidan**)**, sterols, and lipids were the dominant biomolecules in *C. myrica*. Agar and sulfonated polysaccharides were detected in the *G. foliifera* extract*.* Figure 5a-c demonstrates the absorbance spectra of AgNPs capped by *U. rigida, C. myrica,* and *G. foliifera*, which matched the spectra of extracts investigated in the previous section. Absorption bands of extracts at 3394 cm^−1^, 3444 cm^−1^, and 3440 cm^−1^ were redshifted to 3435 cm^−1^, 3424 cm^−1^, and 3440 cm^−1^ for AgNPs capped by *U. rigida*, *C. myrica*, and *G. foliifera*, respectively. The absorption bands at 3280 cm^−1^, 3265 cm^−1^, and 3272 cm^−1^ varied according to the large and small wavenumbers of AgNPs surrounded by the extracts of different algal species. The bands that appeared at 1638 cm^−1^, 1629 cm^−1^, and 1641 cm^−1^ in algae changed after capping. The bands at 1096 cm^−1^, 1032 cm^−1^, and 1044 cm^−1^ red- or blueshifted to 1000 cm^−1^, 1050 cm^−1^, and 1045 cm^−1^ in AgNPs capped with *U. rigida, C. myrica*, *and G. foliifera*, respectively. These changes indicated the electrostatic interaction between AgNPs and functional groups (OH, N–H, and C=O) of the algal species.

**Figure 6:**
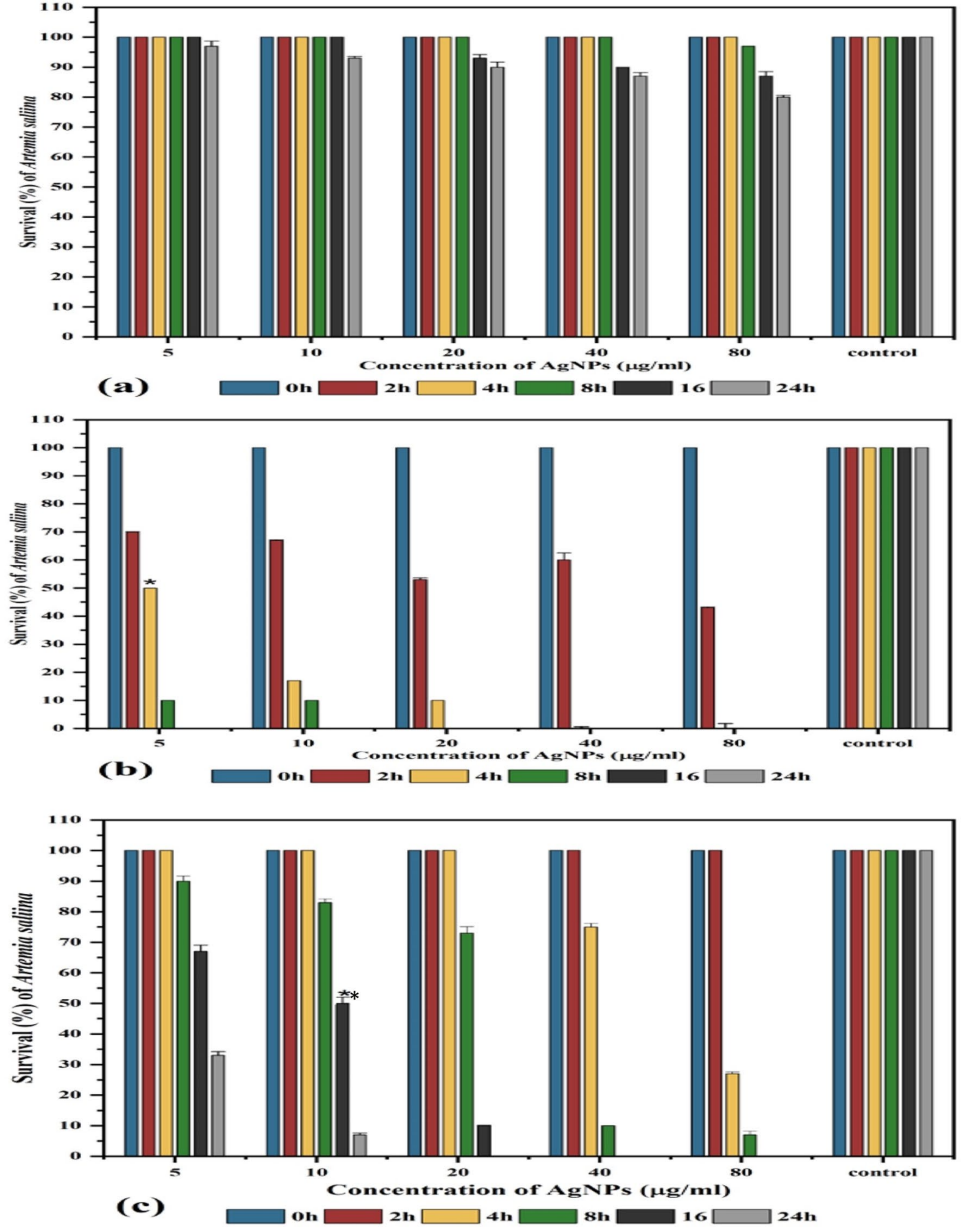
Survival (%) of Artemia salina treated with different concentrations (µg/ml) of biosynthesized AgNPs capped by different marine algal extracts, (**a**) *U. rigida*, (**b**) *C. myrica* and (**c**) *G. foliifera*, and the control (saline solution 3.2% NaCl) containing ten nauplii over 24 h. *= LC50 of *C. myrica* (**b**), **= LC50 of *G. foliifera* (**c**). Each value represents the mean of the sample ± SD (n = 3).

The cytotoxicity and anticancer activities of the biosynthesized AgNPs were assessed using *A. salina* nauplii, normal skin cell lines, and breast cancer cell lines. The cytotoxicity of the AgNPs capped by the biomolecules of different algal species against *A. salina* nauplii is illustrated in Fig. 6. The LC_50_ of *C. myrica-* and G*. foliifera-*based AgNPs against *A. saline* nauplii was 5 μg ml^−1^ and 10 μg ml^−1^ after 4 h and 16 h, respectively, whereas *U. rigida-*based AgNPs did not exhibit cytotoxicity.

**Figure 7:**
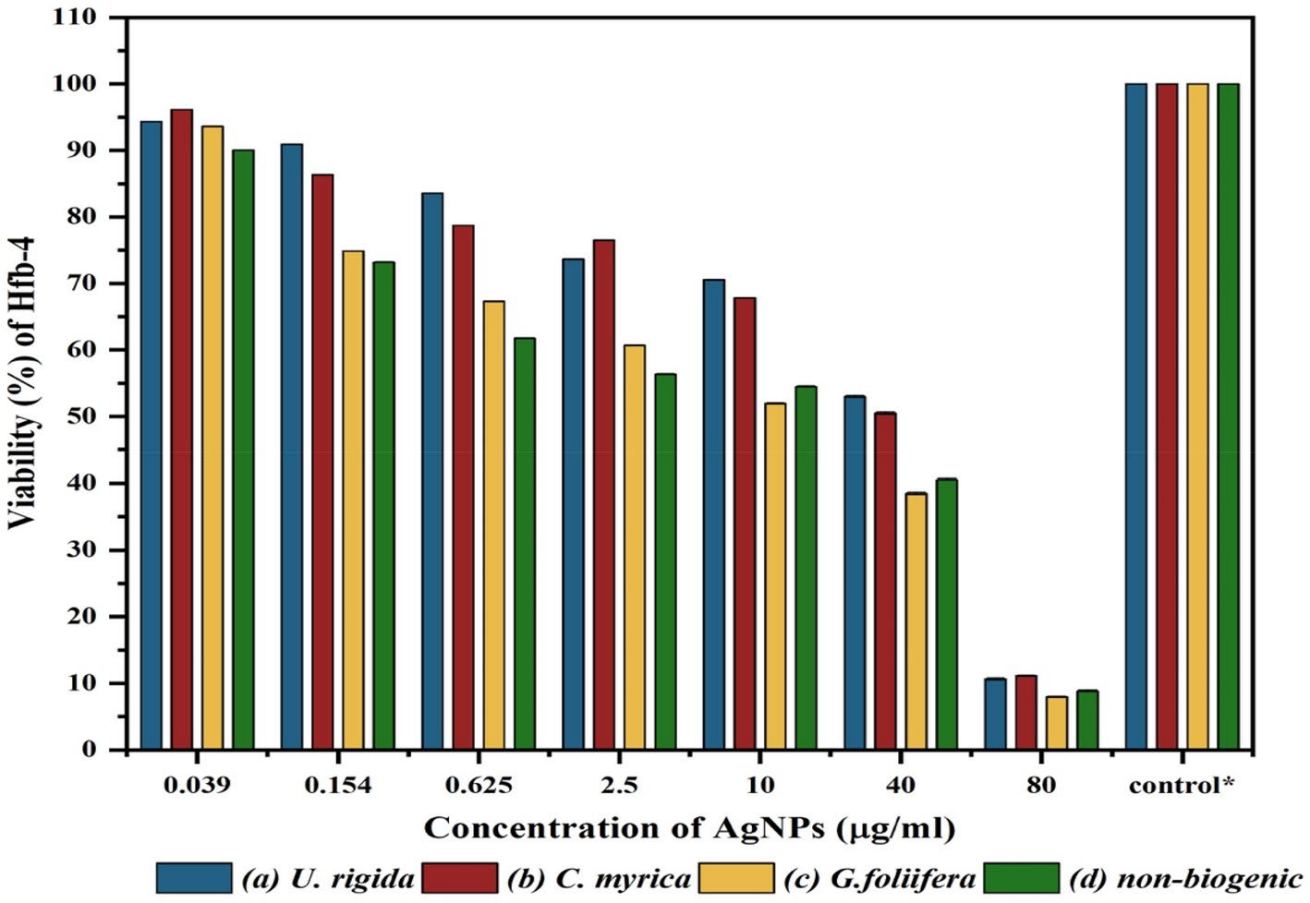
Viability of the human skin cell line (HFb-4) treated with different concentrations (µg/ml) of biosynthesized AgNPs capped by different marine algal extracts, (**a**) *U. rigida,* (**b**) *C. myrica* and (**c**) *G. foliifera,* and (**d**) non-biogenic AgNPs for 24 h. Each value represents the mean of the sample ± SD (n = 3).

**Figure 8:**
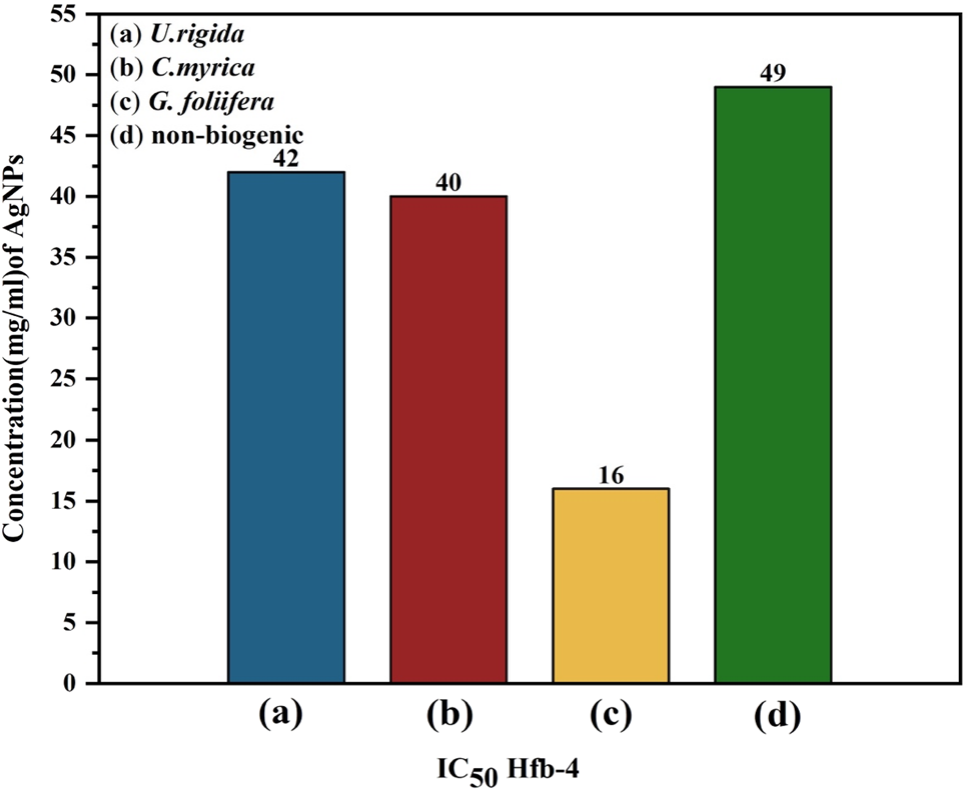
IC50 of the human skin cell line (HFb-4) treated with different concentrations (µg/ml) of biosynthesized AgNPs capped by different marine algal extracts, (**a**) *U. rigida*, (**b**) *C. myrica,* (**c**) *G. foliifera,* and (**d**) non-biogenic AgNPs. Each value represents the mean of the sample ± SD (n = 3).

**Figure 9:**
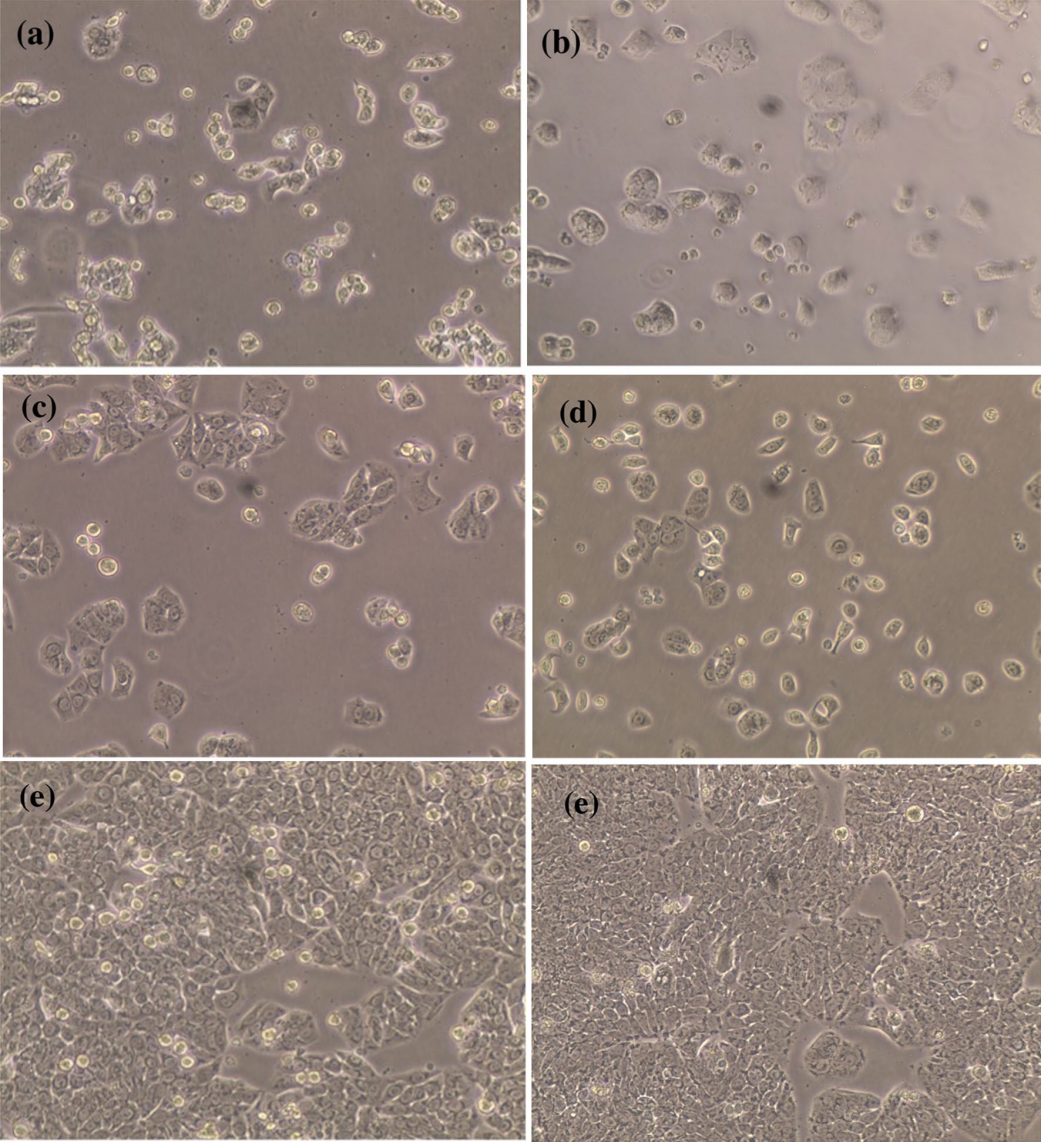
The microscopical visualization (20x) of the human skin cell line (HFb-4) distinguished the variation in the cell viability according to their treatment with biosynthesized AgNPs capped by different marine algal extracts, (**a**) *U. rigida*, (**b**) *C. myrica,* (**c**) *G. foliifera,* (**d**) non-biogenic AgNPs and (**e**) untreated control cells.

HFb-4 cells were also used to determine the cytotoxicity and safety of the AgNPs biosynthesized from different algal species (Fig. 7). The safety of AgNPs to HFb-4 was found to be concentration-dependent, and cell viability gradually decreased with increasing AgNP concentration. In general, compared to the non-biogenic AgNPs, the highest viability of HFb-4 cells were recorded against AgNPs capped with *U. rigida*, followed by those capped with *C. myrica* or *G. foliifera*. These results are similar to the cytotoxicity results of biosynthesized AgNPs against *A. salina* nauplii. Figure 7 reveals that at the initial concentration of AgNPs (0.039 μg ml^−1^), the cell viability was similar among treatments, remaining between 92 and 96%. The viability of HFb-4 cells exposed to 0.154 μg ml^−1^ biogenic AgNPs was 90.9%, 86.3%, 81.0%, and 74.8% for *U. rigida* AgNPs, *C. myrica* AgNPs, non-biogenic AgNPs, and *G. foliifera* AgNPs, respectively. The HFb-4 cell viability was 92.05%, 89.3%, 88.87%, and 67% for *G. foliifera* AgNPs*, U. rigida* AgNPs*, C. myrica* AgNPs, and non-biogenic AgNPs, respectively, when the AgNP concentration was 80 μg ml^−1^. The cytotoxicity level determined as the half-maximal inhibitory concentration (IC_50_) of the biogenic AgNPs capped with different algal species and non-biogenic AgNPs were estimated by the sulforhodamine B (SRB) assay method. The IC_50_ of biogenic AgNPs against HFb-4 cells was 42 µg ml^−1^, 40 µg ml^−1^, and 16 µg ml^−1^ for AgNPs capped with *U. rigida*, *C. myrica,* and *G. foliifera*, respectively, whereas the IC_50_ of non-biogenic AgNPs was 49 µg ml^−1^ (Fig. 8). In addition, the morphological changes of HFb-4 cells treated with the tested AgNPs were microscopically captured as indicated in Fig. 9.

**Figure 10:**
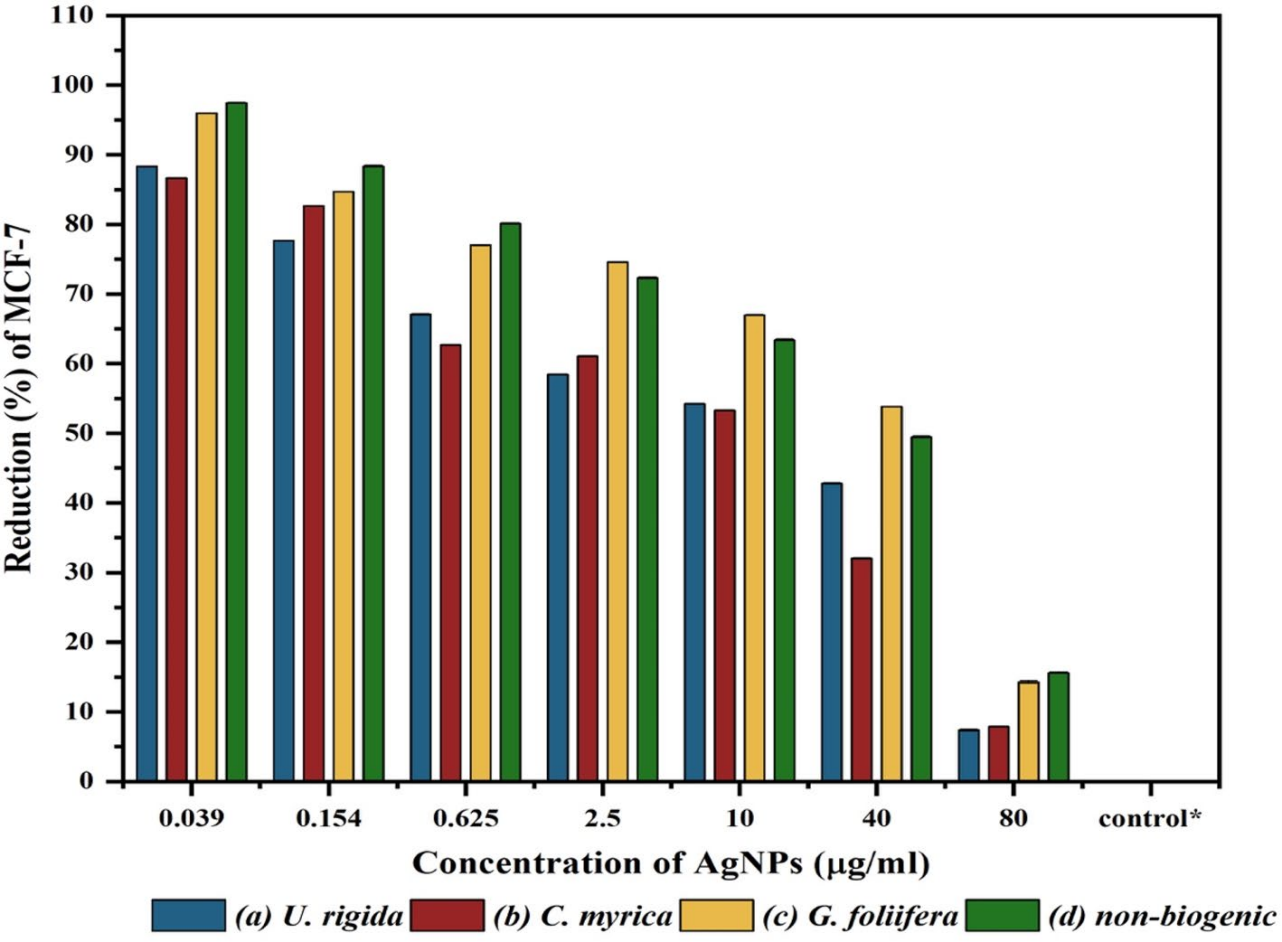
Reduction (%) and anticancer activity of Human Brest cancer cell lines (MCF-7) treated with different concentrations (µg/ml) of the biosynthesized AuNPs capped by different marine algal extracts: (**a**) *U. rigida,* (**b**) *C. myrica* and (**c**) *G. foliifera* and (**d**) nonbiogenic AuNPs during 24 h. Each value represents the mean of the sample ± SD for n = 3.

**Figure 11:**
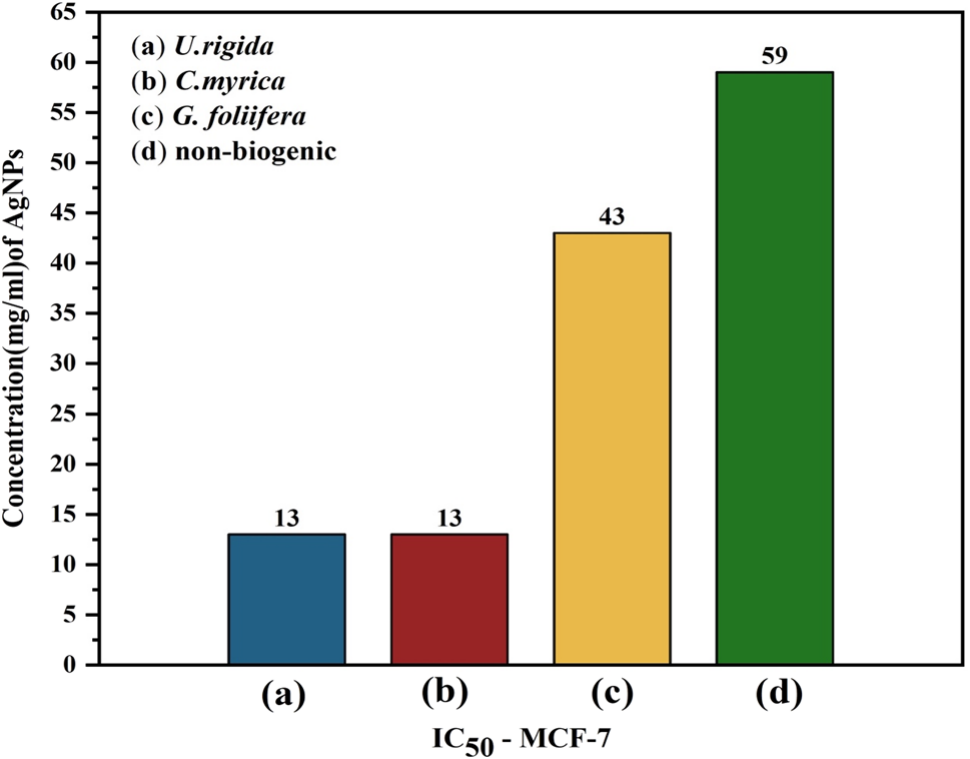
IC50 of the Human Brest cancer cell lines (MCF-7) treated with different concentrations (µg/ml) of biosynthesized AgNPs capped by different marine algal extracts, (**a**) *U. rigida*, (**b**) *C. myrica,* (**c**) *G. foliifera,* and (**d**) non-biogenic AgNPs. Each value represents the mean of the sample ± SD (n = 3).

The anticancer activity of AgNPs synthesized by different algal species was assessed in vitro using human breast cancer cells (MCF-7 cell line) according to the SRB method. The results presented in Fig. 10 indicate that the anticancer activity of the biogenic AgNPs was dose-dependent. AgNPs capped with *U. rigida* extract exhibited significant anticancer activity (92.62%) against the MCF-7 cell line at a concentration of 80 μg ml^−1^, followed by AgNPs capped with *C. myrica* (92.13%) and *G. foliifera* (85.74%). Non-biogenic AgNPs presented the lowest anticancer value of 62%. Interestingly, biogenic AgNPs exhibited 13.33%, 11.64%, and 4.04% anticancer activity against MCF-7 cells, even at a very low concentration of 0.039 µg ml^−1^. Figure 11 shows the cytotoxicity and IC_50_ values of AgNPs of different algal extracts at various concentrations. The IC_50_ values of the biogenic AgNPs were 13 µg ml^−1^, 13 µg ml^−1^, and 43 µg ml^−1^ for *U. rigida, C. myrica,* and *G. foliifera*, respectively, whereas the IC_50_ value of the non-biogenic AgNPs was 59 μg ml^−1^. To validate the selectivity of tested algae mediated AgNPs on breast cancer in relation to normal cells, the selective index was calculated according to SI equation. The SI values were 3.2, 3.07, 0.37, and 0.83 for *U. rigida, C. myrica, G. foliifera*, and non-biogenic respectively.

**Figure 12:**
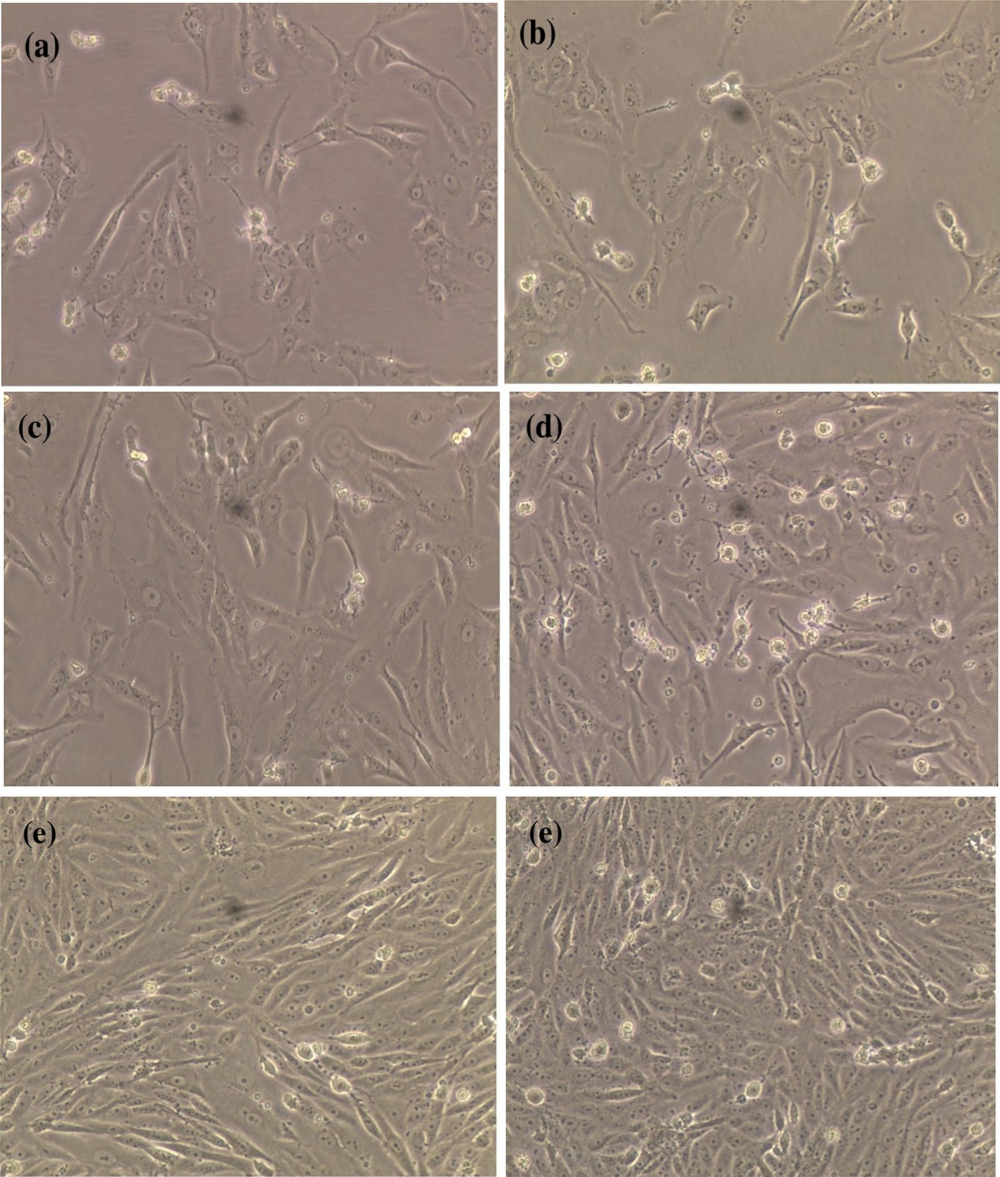
The microscopical visualization (20 ×) of the human breast cancer cell line (MCF-7) distinguished the variation in the cell reduction according to their treatment with biosynthesized AgNPs capped by different marine algal extracts, (**a**) *U. rigida*, (**b**) *C. myrica,* (**c**) *G. foliifera,* (**d**) non-biogenic AgNPs and (**e**) untreated control cells.

To confirm the morphological changes in the MCF-7 cells after treatment with the tested AgNPs, the microscopical images of the cells before and after treatment were captured (Fig. 12). To determine the biomolecules that acted as capping and stabilizing agents during AgNP reduction, FTIR and UV–Vis spectroscopic analyses were performed. The data in Fig. 5 indicate that terpenoids, polyphenols, carotenoids, fatty acids, carbohydrates, and lipids were the dominant biomolecules in the *U. rigida* extract, whereas polyphenols, sulfonated polysaccharides (fucoidan), sterols, and lipids were the dominant biomolecules in *C. myrica*. Compounds such as agar and sulfonated polysaccharides were detected in the *G. foliifera* extract*.*

The antimicrobial activity of the biosynthesized AgNPs was assessed against foodborne pathogenic bacteria such as gram-positive* Bacillus cereus* and *S. aureus*; gram-negative *E. coli*; dermatophyte moulds; and fungi, including *C. albicans*, *T. cataneum,* and *T. mantigrophytes*. The results indicated that the biosynthesized AgNPs exhibited varying antimicrobial activities against the tested pathogens. The highest antimicrobial activity was recorded for the AgNPs capped with the *U. rigida* extract, followed by those capped with *C. myrica* and *G. foliifera* extracts. AgNPs capped with the *U. rigida* extract exhibited the highest antimicrobial activity against *T. mantigrophytes* (40 mm), followed by *T. cataneum* (30 mm) and *E. coli* (19 mm). In contrast, the lowest antimicrobial values were noted for* B. cereus* (14 mm)*, S. aureus* (13 mm)*, C. albicans* (13 mm)*,* and *Cryptococcus neoformans* (11 mm). There was no difference among the results of AgNPs synthesized by ethanolic and aqueous extracts of *U. rigida*. AgNPs synthesized from the *C. myrica* ethanolic extract exhibited the highest antimicrobial activity against *T. mantigrophytes* (30 mm) and *T. cataneum* (30 mm), followed by *B. cereus* (19 mm) and *S. aureus* (17 mm), whereas the lowest values were recorded for *E. coli* (12 mm) and *C. neoformans* (13 mm). AgNPs synthesized with *C. myrica* aqueous extract did not affect *C. albicans* or *T. mantigrophytes* (Table 1).

**Table 1:** Antimicrobial activity of the biosynthesized AgNPs capped by different marine algal extracts, (a) *U. rigida,* (b) *C. myrica* and (c) *G. foliifera*, against tested pathogenic microorganisms indicated by clear zone diameter (CZD^a,b,c^, mm) and minimal lethal concentration (MLC, µg ml^−1^).

*Treatments*	CZD (mm) and MLC (μg ml^−1^)													
	Foodborne pathogenic bacteria						Pathogenic moulds and fungi							
	*B. cereus*		*E. coli*		*S. aureus*		*Cryptococcus neoforrmans*		*C. albicans*		*Trichosporon cataneum*		*T. mantigrophytes*	
	CZD*	MLC	CZD*	MLC	CZD*	MLC	CZD*	MLC	CZD*	MLC	CZD*	MLC	CZD*	MLC
*U. rigida*														
1—AgNPs aqueous	16 ± 1	64	12 ± 0.6	64	14 ± 1	64	11 ± 0	64	12 ± 0.6	64	29 ± 0.6	32	18 ± 0	32
2—Aqueous extract	0.0	–	0.0	–	0.0	–	0.0	–	0.0	–	18 ± 1	–	12 ± 0	1024
3—AgNPs Ethanol	14 ± 1	64	19 ± 1	64	13 ± 1	64	11 ± 0	64	13 ± 0	64	30 ± 0	32	40 ± 0	32
4—Ethanolic extract	0.0	–	0.0	–	0.0	–	0.0	–	0.0	–	18 ± 1	–	11 ± 0	1024
5—Control	0.0	–	0.0	–	0.0	–	0.0	–	0.0	–	0.0	–	0.0	-
*C. myrica*														
1—AgNPs aqueous	19 ± 1	64	13 ± 1	64	17 ± 1	64	13 ± 0.6	64	0.0	–	19 ± 0.6	64	0.0	-
2—Aqueous extract	0.0	–	0.0	–	0.0	–	0.0	–	0.0	–	0.0	–	0.0	-
3—AgNPs Ethanol	17 ± 1	64	12 ± 0	64	15 ± 1	64	13 ± 0.6	64	15 ± 1	64	30 ± 0	32	30 ± 0	32
4—Ethanolic extract	0.0	–	0.0	–	0.0	–	0.0	–	0.0	–	16 ± 1	1024	0.0	-
5—Control	0.0	–	0.0	–	0.0	–	0.0	–	0.0	–	0 ± 0	–	0.0	-
*G. foliifera*														
1—AgNPs aqueous	14 ± 1.5	64	11 ± 0	64	18 ± 0.6	32	14 ± 0.6	64	13 ± 0	64	18 ± 0	64	0.0	-
2—Aqueous extract	0.0	–	0.0	–	0.0	–	0.0	–	0.0	–	0.0	–	0.0	-
3—AgNPs Ethanol	13 ± 1	64	14 ± 1	64	17 ± 0.6	32	13 ± 0.6	64	0.0	–	17 ± 0	64	23 ± 0	64
4—Ethanolic extract	0.0	–	0.0	–	0.0	–	0.0	–	0.0	–	0.0	–	0.0	-
5—Control	0.0	–	0.0	–	0.0	–	0.0	–	0.0	–	0.0	–	0.0	-
*Antibiotics for comparison*														
Fluconazole (100 μg ml^−1^)	Nd	nd	nd	nd	– nd	nd	28	nd	30	nd	28	nd	35	nd
Chloramphenicol (30 µg ml^−1^)	18	nd	20	nd	0.0	nd	nd	nd	nd	nd	nd	nd	nd	nd
Augmentin (30 µg ml^−1^)	0.0	nd	17	nd	0.0	nd	nd	nd	nd	nd	nd	nd	nd	nd
Gentamycin (30 µg ml^−1^)	19	nd	10	nd	18	nd	nd	nd	nd	nd	nd	nd	nd	nd

^a^Each value represents the mean of the sample ± SD (n = 3). ^b^The diameter of the inhibition zone was measured as the clear area centred on the agar well containing the sample. ^c^nd, not determined.

AgNPs synthesized with *G. foliifera* (both ethanolic and aqueous extracts) comparatively exhibited mild effects against most of the tested microorganisms. The data in Table (1) also show that the minimum lethal concentrations (MLCs) of AgNPs capped by different algal species were detected at very low concentrations in the range of 64–128 μg ml^−1^. Furthermore, antimicrobial activity was not detected in most of the Ag-free ethanolic and aqueous algal extracts at low concentrations. A small, unstable clear zone was only recovered at a higher dose of 200 mg ml^−1^ Ag-free ethanolic and aqueous algal extracts after a relatively long incubation period (data not shown). The antimicrobial activity of biogenic AgNPs was generally either equal to or higher than that recorded against antibiotics used in this study.

## Discussion

During this study, aqueous algal extracts were utilized to reduce silver nitrate (AgNO_3_) to a brownish-yellow colour, which indicates the formation of AgNPs. The phenomenon of surface plasmonic resonance (SPR) contributes to the distinct brownish-yellow colour reported by Link and El-Sayed^23^. The excitation and reduction of AgNO_3_ might be the reason behind the appearance of SPR^24^. The colour of the control AgNO_3_ solution (without algal extract) did not change. The algal-mediated AgNP synthesis mechanism is known to induce electrostatic interactions between silver anions and functional groups of the algal extract. Algal phytochemicals include hydroxyl and amino functional groups, which serve as effective metal-reducing and capping agents to provide a robust coating on metal NPs in a single step^25^. UV–Vis spectra revealed that AgNPs capped by *U. rigida* had a higher wavelength, representing larger particles than those capped by *C. myrica* and *G. foliifera*. The presence of more functional groups in the chemical structure of* U. rigida* might have contributed to this redshift, as reported by Kannan et al.^26^. Proteins, carbohydrates, phenols, lipids, free amino acids, chlorophyll a and b, fatty acids, carotenoids, and flavonoids facilitated the increase in AgNP size during formation. The smaller shift in the absorbance peak of AgNPs capped with *G. foliifera* at 415 nm might be due to the presence of only a few functional groups (alkaline, agar, pyruvic acid, and sulfate compound polysaccharides), as reported by El-Kassas and Komi^27^. These results concluded that the presence of more functional groups in algae allows for Ag^+^ ions to aggregate more readily with each other to increase their size. The UV–vis findings of this study are different than those of previous reports^27–30^. TEM images also depicted different AgNP sizes and shapes compared to previously reported studies^27,29,31,32^. This result might be due to the different algal species that were collected under different environmental conditions of sampling sites in the Red Sea. FTIR analysis tentatively identified bands based on the reference standards and published FTIR spectra related to specific molecular groups^33,34^. The FTIR results confirmed that the hydroxyl functional group of polyphenols might be involved in the bioreduction of Ag(I) ions into Ag(0). The carbonyl group of proteins also possesses a relatively strong ability to bind metal. Therefore, most likely, the proteins or enzymes capped the AgNPs to prevent the agglomeration of particles, and hydroxyl groups of the secondary metabolites of algal material might be involved in AgNP synthesis^35^. Similarly, Vijayaraghavan et al.^36^ revealed that the hydroxyl groups present in brown algal polysaccharides were involved in the bioreduction of Ag(I) ions into Ag(0). Srivastava et al.^37^ reported that protein biomolecules of algal extracts perform a dual function of reducing silver ions and stabilizing AgNPs in aqueous medium. They also stated that active chemical groups play a crucial role in reducing metallic ions and the subsequent formation of nano/microparticles during biogenic synthesis. Based on the surface chemistry of the abovementioned AgNPs, it can be concluded that polyphenols, peptides, and proteins of the studied algal extracts were the key biomolecules engaged in the dual function of Ag(I) reduction and sufficient capping of the AgNPs. The positively charged groups of algal biomolecules might have regulated the surface-mediated process through electrostatic interactions, which could have begun the nucleation of Ag(0) to Ag atoms, finally forming AgNPs. The results suggested that molecules attached to AgNPs have free and bound hydroxyl, carboxyl, and amide groups. Furthermore, amide groups may also be present in the aromatic rings. This result indicates that these compounds could be polyphenols with aromatic rings and bound amide regions. Therefore, the capping ligand of the AgNPs can be either an aromatic compound or amine. The literature survey presents marine seaweeds as a rich source of phenolic compounds, especially bromophenols. The biological properties of polyphenols include antioxidant^38^, and anticancer^39^ effects. Furthermore, tannins and flavonoids are naturally occurring seaweed polyphenolic compounds that are only found in marine algae^40^. Algae also contain high amounts of polyphenols such as catechin, gallic acid, epicatechin, and epigallocatechin gallate^41^. A broad range of biological activities is exhibited by natural algal products, including cytotoxic and antimitotic activities. The electrostatic interaction between AgNPs and different functional groups (OH, N–H, and C=O) of various algal species extracts might change the algal and algal-mediated AgNP spectra. These results are in agreement with previous reports^28,31,42,43^. FTIR and UV–Vis spectroscopic analyses determined the possible involvement of *U. rigida, C. myrica,* and *G. foliifera* in reducing and stabilizing AgNPs.

The cytotoxicity results of AgNPs capped with biomolecules of different algal species indicated that AgNPs synthesized with *U. rigida* extracts did not pose cytotoxic effects against *A. salina*, whereas the cytotoxic effects of *C. myrica* and *G. foliifera* were noted to be directly proportional to the increase in AgNP concentration. To calculate the IC_50_ values, *A. salina* nauplii mortality was recorded against different concentrations (5–80 μg ml^−1^) of biosynthesized AgNPs, and the lethality was found to be directly proportional to the AgNP concentration as confirmed by the microscopical visualization of treated and untreated cells. In this study the *U. rigida* (SI = 3.2) and *C. myrica* (SI = 3.07) were more selective cytotoxic on breast cancer in relation to normal cells than *G. foliifera* and non-biogenic. These results are in line with the reported cytotoxicity of biogenic NPs of different seaweeds, such as *Turbinaria conoides*^44^, *U. rigida* ^45^, *Sargassum boveanum*^46^, *Oscillatoria sp*.^47^, *Cladophora fasciatus*^48^, and *Sargassum ilicifolium*^49^. Therefore, it could be concluded that the toxicity of the studied biosynthesized AgNPs is dose-dependent. Cancer contributed to one in every six deaths during 2018 and is considered among the leading causes of global human mortality. However, most cancer-related deaths (70%) occur in middle- and low-income countries^50^. The current therapeutic strategies for cancer also have many side effects with limitations in treating specific types of cancer^51^. Therefore, the development of new anticancer therapeutic agents with limited side effects is required to improve the current therapeutic strategies^7,52^**.** During this study, AgNPs synthesized by *U. rigida* and *C. myrica* exhibited significant anticancer activity (93%) against MCF-7 cells, followed by *G. foliifera* (85.74%) as confirmed by the microscopical visualization of treated and untreated cells. In contrast, the non-biogenic AgNPs presented the lowest anticancer value of 62%. Similarly, AgNPs synthesized by *U. rigida* and *C. myrica* demonstrated the lowest cytotoxicity against HFb-4 cells. These results contradict the anticancer activity of AgNPs synthesized by *U. rigida* and *C. myrica*, where the highest reduction in viability was observed in MCF-7 cells. However, the severe reduction in viability in MCF-7 cells might be due to enhanced cellular uptake and retention of NPs. NPs can enter cells via endocytosis due to their small size without facing efflux by P-glycoprotein^53^. The dependence of AgNP anticancer activity on their size, shape and concentration has been reported. NPs are safe at relatively low doses, but higher doses can be toxic. Cytotoxicity or anticancer activity may vary against different formulations of various AgNPs^54^. The AgNPs synthesized during this study by algal extracts of *U. rigida*, *C. myrica,* and *G. foliifera* were almost spherical, and their size ranged between 12 and 24 nm. These AgNPs exhibited significant anticancer activity (93%) against breast cancer cell lines. However, the anticancer mechanisms of AgNPs are unclear and complicated. It has been proposed that AgNPs can inhibit the growth of tumour cells by destroying the cellular ultrastructures that induce reactive oxygen species (ROS) production and DNA damage^4^. Pei et al.^12^ reported that DNA damage could change the gene expression that leads to apoptosis. Interestingly, ROS production and subsequent damage resulting from oxidative stress are dependent on AgNP size, as smaller NPs induce the overproduction of ROS. AgNPs can disrupt the mitochondrial chain and complex, which leads to superoxide anion leakage^55^. Furthermore, AgNPs can inactivate proteins, regulate signalling pathways or block tumour cell metastasis by inhibiting angiogenesis within lesions to induce tumour cell apoptosis^56^. FTIR and UV–Vis spectroscopic data indicated that terpenoids, polyphenols, carotenoids, sulfonate polysaccharides (fucoidan), carbohydrates, sterols, lipids, and agar possibly reduced AgNPs and acted as capping, stabilizing, antimicrobial, and anticancer agents. Similar studies have also detected polyphenols, fatty acids, polysaccharides, ionic trace minerals, vitamins, flavonoids, amino acids, fucoidan, enzymes, lipids, terpenoids, chlorophylls, alkaloids, carbohydrates, pyruvic acid, and aliphatic fluoro compounds in *U. rigida*, *C. myrica* and *G. foliifera* aqueous extracts, which might act as anticancer and antimicrobial agents^42,57^.

To counter the increasing resistance of pathogenic microbes against conventional antibiotics, safe, effective, stable, and highly efficient alternatives should be explored. Green synthesis of AgNPs is a safe and eco-friendly alternative to conventional antibiotics^9^ .This study also evaluated the antimicrobial activity of biosynthesized AgNPs capped by the bioactive compounds of different algal species, including *U. rigida, C. myrica*, and *G. foliifera*, against the gram-positive bacteria *B. cereus* and *S. aureus*; the gram-negative bacterium *E*. *coli*; and dermatophytic fungi, including *C. albicans*, *C. neoformans*, *T. cataneum,* and *T. mantigrophytes*. Similar study reported that AgNPs synthesized by the aqueous leaf extract of *Ardisia solanacea* exhibited moderate activity against food borne pathogens and *Trichophyton mentagrophytes* (5).

The biosynthesized AgNPs depicted variable levels of broad-spectrum antimicrobial properties against the tested pathogens. Dermatophytic fungi were found to be more sensitive than foodborne bacteria. AgNPs synthesized with *U. rigida* exhibited the highest antimicrobial activity, followed by those synthesized with *C. myrica* and *G. foliifera*. The highest antimicrobial activity was noted against *T. mantigrophytes*, followed by* T. cataneum* and *E. coli*, whereas the lowest values were recorded for* B. cereus, S. aureus, C. albicans,* and *C. neoformans*. The presence of different bioactive constituents among algal species might have contributed to the varying antimicrobial activity of algal NP extracts, as suggested by Cox et al.^58^. These authors reported that secondary metabolites such as polyphenols, terpenes, acetogenins, and aromatic compounds produced by brown and red macroalgae possess several biological activities, including anti-inflammatory and antibiotic properties. The difference in the thicknesses of cell walls of gram-positive and gram-negative bacteria can be another reason for the varying antimicrobial activity of AgNPs against pathogens^59^. Factors such as the size, shape, concentration, time, and charge of AgNPs can also impact the antibacterial activity^60^. Dermatophytosis is caused by some yeast and fungi, including *C. albicans*, *Trichophyton sp*., and *Trichosporon*. The limited number of effective antifungal drugs, toxicity level of the available antifungal drugs, relapse of *Candida* infections, resistance of *Candida* to commonly used antifungal drugs, and high cost of antifungal drugs can hinder candidiasis treatment^61,62^. AgNPs synthesized by different algal extracts during this study exhibited significant antifungal activity against *Trichophyton sp*. and *Trichosporon*, which are associated with skin infections and other health problems. The antifungal activities of the biosynthesized AgNPs against *T. mantigrophytes* and *T. cataneum* were detected through the formation of clear inhibitory zones, which revealed complete growth inhibition. These results were confirmed by determining the minimal lethal concentration of AgNPs, which ranged from 64–128 μg ml^-1^. It is worth mentioning that antimicrobial activity was not detected in the control Ag-free ethanolic or aqueous algal extracts, reflecting that the antimicrobial activity was directly related to the AgNPs. Relatively high doses of algae or medicinal plant extracts have been effectively used against bacterial growth. Therefore, it is important to explore an alternative method to maximize the efficacy of extracts. The use of algal extracts for synthesizing NPs has emerged as an effective alternative to chemical synthesis methods. A significant role of NPs has been reported in various scientific fields^11,63^. The mechanism of AgNPs as antimicrobial agents is not clearly known and is still a debated topic. In this context, it has been proposed that AgNPs can inhibit bacterial growth or kill bacteria by inducing membrane destruction, DNA damage, ROS generation, protein denaturation, and enzyme inactivation^64,65^. Similarly, AgNPs can saturate and adhere to fungal hyphae and produce insoluble compounds to inactivate sulfhydryl groups of the fungal cell wall, disrupt the membrane, and bind lipids and enzymes, leading to cell lysis^66^.

## Conclusion

In conclusion, AgNPs were biosynthesized using marine algal extracts of *U. rigida*, *C. myrica*, and *G. foliifera* as reducing and capping agents. Surface plasmonic bands of the biosynthesized AgNPs revealed an almost spherical shape of AgNPs with sizes of 12 nm (*U. rigida*), 17 nm (*C. myrica*), and 24 nm (*G. foliifera*). FTIR results suggested that the molecules were attached to AgNPs through OH, C=O, and amide groups in marine algal extracts. The major constituents detected in algal extracts included proteins, terpenoids, polysaccharides, chlorophylls, fatty acids, amides, flavonoids, polyphenols, sulfate compounds, carotenoids, agar groups, lipids, and aliphatic fluoro compounds. This is probably the first report emphasizing that AgNPs synthesized by marine algal species, particularly AgNPs prepared with *U. rigida*, could be considered an effective alternative against dermatophytic fungi associated with skin infections. Moreover, the AgNPs also exhibited significant anticancer potential against cancerous MCF-7 cells, especially *U. rigida* and *C. myrica* without causing cytotoxicity against *A. salina* nauplii. However, in vivo studies are required to further elaborate their anticancer efficacy through animal models. Challenges related to biosynthesized AgNPs, including genotoxicity, therapeutic window, safety profile, pharmacokinetics, and antibacterial resistance, should also be addressed in future studies.

## Materials and methods

### Preparation of algal extracts

Different marine algal species were collected from the Red Sea coast, cleaned, and transported to the laboratory in ice bags. Algae were washed several times with distilled water and dried in the shade at room temperature for 1 week. The dried algae were ground into a fine powder, sieved (> 0.5 mm), and kept at 4 °C until use. To prepare the aqueous extract, 10 g of each algal powder was mixed with 400 ml of deionized water and heated to 70 °C for 15 min. Algal precipitates were filtered three times through Whatman paper No. 1 until clear extracts were obtained. The clear extracts were lyophilized and stored at 4 °C until use^67^. To prepare the ethanolic extract, dried powder (10 g) of each algal species was soaked in 200 ml of ethanol (95%) and shaken at room temperature for 24 h. Then, the mixture was filtered through Whatman paper No. 1 and evaporated to dryness in a rotary evaporator at 45 °C. Stock solutions (200 mg/ml H_2_O) of each algal extract were prepared for further work.

### Synthesis of AgNPs using different marine algal extracts

The synthesis of AgNPs from marine algal extracts was carried out according to Isaac and Renitta^67^. Briefly, 80 ml of 10^–3^ M silver nitrate (AgNO_3_, Sigma Aldrich) solution was prepared and added to 20 ml of each algal extract inside a conical flask. The mixture was stirred on a hot plate at 70 °C for 15 min in a heating mantle to reduce the metal ions. The reduction reaction was catalysed by adding 1 ml of fresh sodium borohydride (0.03783 g 10 ml^−1^ H_2_O). Subsequently, the biosynthesized AgNP solutions were gently mixed, cooled, and stored at room temperature until use.

### Synthesis of non-biogenic AgNPs

Ascorbic acid and polyvinyl pyrrolidone (PVP) were used as a reducer and preservative, respectively, to synthesize non-biogenic AgNPs^68^. PVP (0.8 g) was added to 80 ml of ascorbic acid solution (5 × 10^–4^ mol l^−1^) and stirred for 20 min at room temperature. Then, 1.4 ml of NaOH solution (5 × 10^–4^ mol l^−1^) was added and stirred for 5 min. Finally, 0.8 ml of AgNO_3_ (0.1 mol l^−1^) was added to the solution and vigorously stirred at 80 °C for 30 min in a water bath. The Ag^+^ concentration in the non-biogenic AgNPs was estimated to be 80. The final colloidal, non-biogenic AgNP solution was kept at room temperature until use.

### Physiochemical characterization of biosynthesized AgNPs

Spectral analysis of the biosynthesized AgNPs capped with different algal extracts was carried out by measuring the absorption spectral maxima in a UV–Vis Thermo-scientific Evolution 220 Spectrophotometer at a resolution of 2 nm. The absorption spectral maxima were scanned at wavelengths of 200–900 nm.

The distributions, shape, and particle size of the NPs were studied using a JEOL JEM-1100 microscope (JEOL Ltd., Tokyo, Japan) equipped with a tungsten thermionic gun operating at an accelerating voltage of 100 kV. A CCD camera captured TEM images. NPs were dispersed in the solution for approximately 5 min using an ultrasonication device, and a droplet was immediately placed on a copper grid and dried at room temperature to obtain TEM measurements^45^.

To analyse the biosynthesized AgNPs with FTIR spectroscopy, some of the NP solutions were centrifuged at 15,000 rpm for 15 min. The precipitated product was washed with deionized water to eliminate the free proteins/enzymes that were not capping the NPs. The purified suspension was freeze-dried to obtain a dry powder that was analysed using FTIR. The samples were dried and ground with KBr pellets. A disk of 50 mg of KBr was prepared with a mixture of 2% finely dried specimens and examined using a Jasco Model 300E FTIR spectrometer. The potential functional groups responsible for the reduction and capping behaviour of biomolecules were detected by analysing the spectra of algal extracts and synthesized AgNPs in the wavenumber range of 500–4000 cm^−1^ ^69^.

### Cytotoxicity assessment of biosynthesized AgNPs against brine shrimp (*Artemia salina*)

Brine shrimp (*A. salina*) cyst larvae were prepared and maintained under laboratory conditions to determine the cytotoxicity of synthesized AgNPs. *A. salina* cysts (0.75 g) were suspended in 100 ml of saline solution (NaCl 3.2%) inside a 500 ml glass jar. Larval hatching was carried out by continuous aeration (JBL Artemio Set for breeding Artemia Brine) under an incandescent lamp (40–60 watts) at 25–29 °C for 36 h. After hatching, active and free-floating nauplii were collected from bright illumination and kept until used for cytotoxicity analysis. Serially diluted concentrations (5–80 µg ml^−1^) of AgNPs were used against *A. salina* larvae to determine the cytotoxicity of biosynthesized AgNPs capped with different algal extracts (*U. rigida*, *C. myrica,* and *G. foliifera*). Freshwater was used as a negative control (without AgNPs). Ten shrimp larvae were transferred into a 5 ml solution for each treatment and stored at 25 °C for 24 h in the dark. Finally, the number of dead and alive larvae were determined with a microscope, and LC_50_ values were determined according to Azizi et al.^70^.

$${\text{Mortality}}\% \, = \,\sum {\text{ dead\,test\,larvae}}\, - \,\sum {\text{ dead\,control\,larvae}}/\sum {\text{ test\,larvae}}\, \times \,{1}00.$$

### Cytotoxicity assessment of biosynthesized AgNPs against skin cell line (Hfb-4)

The media contained 10% antibiotic-free Fetal Bovine Serum. FBS (Sigma, USA), 100 mg penicillin, and 2 mg streptomycin. The cells were kept at 37 °C with 95% relative humidity and 5% CO2. The cytotoxicity of biosynthesized AgNPs against skin cell lines (HFfb-4) was assessed using 96-well plates^71^. To test the viability of HFfb-4 cells after 24 h, then incubated with and without (control) of serial dilutions (0–80 μg/ ml) of algal-capped synthesized AgNPs and non-biogenic AgNPs for 24 h (37 °C dark). Then, 0.5 mg/ml MTT was added to cells and incubated for 4 h, the formazan crystals dissolved in 200 μl DMSO. After reduction, tetrazolium salt becomes a brightly colored product that can be measured colorimetrically 570 nm. The cytotoxicity of AgNP was calculated as follows:

$${\mathrm{Mean\,Absorbance\,of\,sample}}/{\mathrm{Absorbance\,of\,control }} \times 100.$$

### Anticancer activity of biosynthesized AgNPs against human breast cancer cell line (MCF-7)

Anticancer activity of Biosynthesised AgNP evaluated in a human breast cancer cell line (MCF-7) Skehan et al.^72^ evaluated the anticancer activity of biosynthesized AgNPs capped with different algal extracts (*U rigida, C myrica, and G foliifera*) using human breast adenocarcinoma cell line (MCF-7) cells. Anti-cancer activity was tested against MCF-7 cells using the sulforhodamine B (SRB) method. Cells were incubated at 37 °C in a CO2 incubator for 24 h to allow the cells to attach to the plates. On the other hand, the MCF-7 cells were added to wells with serially diluted biosynthesized and non-biogenic AgNPs (0–80 µg/ml) for 48 h. The cells were fixed in 50 ml 50% trichloroacetic acid at 4 °C for 11 h. The plates were then washed in distilled water and stained with 50 ml of 0.4% SRB dissolved in 1% acetic acid for 30 min at room temperature. To remove excess dye, the cells were washed 4 times with 1% acetic acid. Solubilized in 100 ml (10 mmol-1 Tris-base) (pH 10.4), the optical density of each well was measured at 570 nm. The IC50 was calculated using master ples-2010^73^. Morphological changes in the MCF-7 cells before and after treatment with the tested AgNPs were captured using Olympus CKX3 / 41 inverted microscope (20 × magnification). The cell viability percentage was computed as follows:

$${\mathrm{Mean\,Absorbance\,of\,sample}}/{\mathrm{Absorbance\,of\,control}}\,{1}00.$$

### Selective-index

To determine the cytotoxic selectivity of the substances tested, the selectivity index (SI) was calculated according to the following Eq. ^74^:

$$\mathrm{SI}=\frac{\mathrm{ IC}50\mathrm{ \,no\, cancer\, cells}}{\mathrm{IC}50\mathrm{\,cancer\, cells}}.$$

### Determination of antimicrobial activity and minimal lethal concentration of biosynthesized AgNPs

The antimicrobial activity of the biosynthesized AgNPs was determined by using gram-positive *B. cereus* ATCC11778 and *S. aureus* (ATCC25923) and gram-negative *E. coli* (ATCC25922). The activity of biosynthesized AgNPs was also assessed against moulds and dermatophytic fungi such as *T. mantigrophytes*, *C. albicans* (ATCC 10,231), and *T. cataneum*. All fungi were obtained from Fungi Center, Assiut University, Egypt. Bacterial stock cultures were maintained on nutrient agar slants at 4 °C, while fungi and *Candida* yeast were maintained on potato dextrose agar (PDA) slants at 4 °C.

The antimicrobial activity of the biosynthesized AgNPs was assessed by the agar well diffusion method according to Elbanna et al.^62^. Briefly, sterilized Mueller–Hinton agar (used for bacteria) and PDA (used for fungi and moulds) were poured into sterilized Petri dishes and solidified at room temperature. The agar plates were swabbed from overnight bacterial or fresh fungal cultures. A sterile cork borer (9 mm) was used to make the wells at the centre of agar plates, and 200 µl of the biosynthesized AgNPs was transferred to the wells. Plates with pathogenic bacteria or *C. albicans* were incubated at 37 °C and 30 °C for 24 h, respectively. Simultaneously, pathogenic fungi were incubated at 30 °C for 48–72 h. The diameter of clear zones (mm) around each well determined the antimicrobial activity. Water without test compounds was used as a control. The antibacterial activity of antibiotics was assessed through the agar disk diffusion method according to Bauer and AW^75^ by measuring the diameter (mm) of clear zones around each well. Gentamycin (30 µg), chloramphenicol (30 µg), augmentin (30 µg), and fluconazole (100 μg ml^−1^) served as comparative standards in antibacterial and antifungal tests, respectively.

### Determination of minimal lethal concentration of biosynthesized AgNPs

The MLCs of biosynthesized AgNPs against pathogenic microorganisms were assessed by the serial fold dilution method according to Jobran^76^. Serially diluted concentrations (0–80 µg ml^−1^) of biosynthesized AgNPs were pipetted into tubes containing 4 ml of LB or PD broth medium for pathogenic bacteria or fungi, respectively. Each tube was inoculated with 0.4 ml (0.5 McFarland medium) of standardized bacterial suspension of test species containing 1 × 10^6^ cell ml^−1^. In the case of pathogenic fungi, serially diluted concentrations of biosynthesized AgNPs were pipetted into tubes containing 4 ml of PD broth, and each tube was inoculated with 1 × 10^6^ prepared spores. All inoculated tubes were incubated under appropriate conditions for each microorganism. After the incubation period, a 0.1 ml sample from each tube was subcultured on LB agar or PDA plates and incubated under appropriate conditions for each microorganism. The lowest concentration of biosynthesized AgNPs producing a viable count of less than 0.1% of the original inoculum (1 × 10^6^ cell ml^−1^) was assumed to be the MLC.

## Supplementary Information

Supplementary Information 1.

Supplementary Information 2.

## Data Availability

All data are contained within the article.
