# Supplementary material for: Anticancer and antimicrobial activity of biosynthesized Red Sea marine algal silver nanoparticles
Source: Sci Rep. 2022 Feb 14;12:2421. doi: 10.1038/s41598-022-06412-3 (PMC8844081; doi:10.1038/s41598-022-06412-3)
Supplement: Supplementary file 1 — Supplementary Information 1. [file 41598_2022_6412_MOESM1_ESM.docx]

*******Supplementary data**

**Supplementary (1) UV.vis Extract of different algal species and assay cytotoxicity**


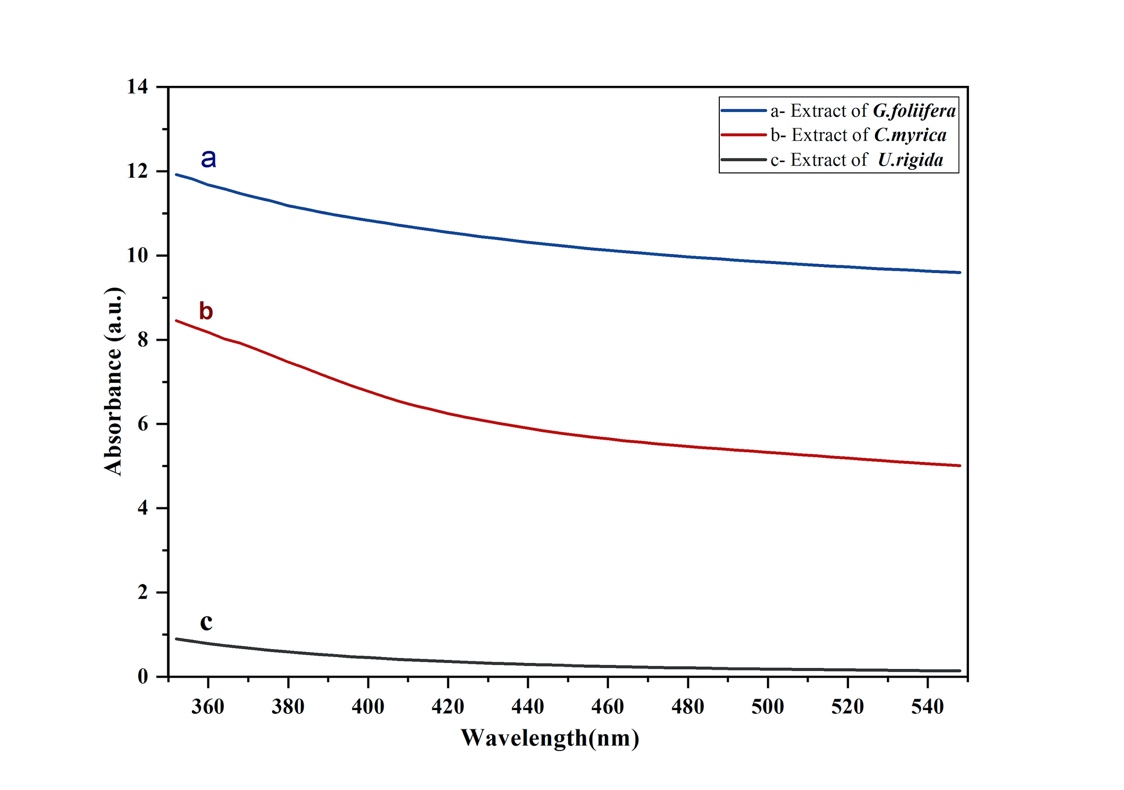


**Figure.** UV-vis spectra of the different marine algal extracts: (a) *U. rigida,* (b) *C. myrica* and (c) *G. foliifera***.**

**Supplementary (2)**

- Video summarized and reveal cytotoxicity assay of algae-mediated AgNPs against *Artemia salina* nauplii :

[**https://streamable.com/lkadsd**](https://streamable.com/lkadsd)

**
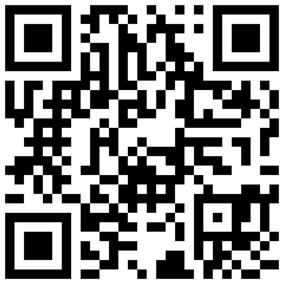
**
