# Supplementary material for: Anticancer and antimicrobial activity of biosynthesized Red Sea marine algal silver nanoparticles
Source: Sci Rep. 2022 Feb 14;12:2421. doi: 10.1038/s41598-022-06412-3 (PMC8844081; doi:10.1038/s41598-022-06412-3)
Supplement: Supplementary file 2 — Supplementary Information 2. [file 41598_2022_6412_MOESM2_ESM.docx]

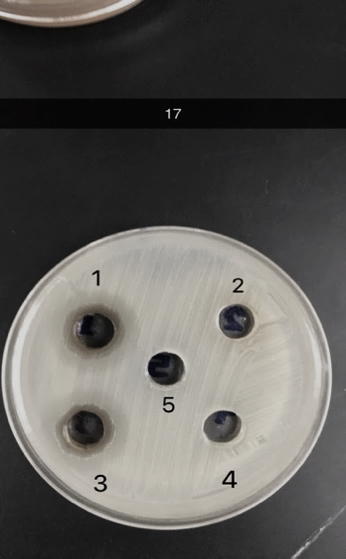


AgNPs

*Cystoseira myrica*

*Bacillus cereus*

Figure (A) Antimicrobial activity of the biosynthesized AgNPs capped by *Cystoseira myrica*, against tested pathogen microorganisms *Bacillus cereus* indicated by clear zone diameter (CZD, mm). (1) AgNPs aqueous, (2) Aqueous extract, (3) AgNPs Ethanol extract, (4) Ethanol extract and (5) Control.


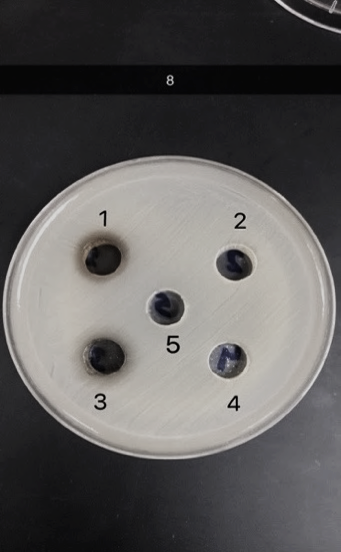


AgNPs

*Ulva rigida*

*Escherichia coli*

Figure (B) Antimicrobial activity of the biosynthesized AgNPs capped by *U. rigida*, against tested pathogen microorganisms *Escherichia coli* indicated by clear zone diameter (CZD, mm). (1) AgNPs aqueous, (2) Aqueous extract, (3) AgNPs Ethanol extract, (4) Ethanol extract and (5) Control.

Figure (C) Antimicrobial activity of the biosynthesized AgNPs capped by *C. myrica*, against tested pathogen microorganisms *Staphylococcus aureus* indicated by clear zone diameter (CZD, mm). (1) AgNPs aqueous, (2) Aqueous extract, (3) AgNPs Ethanol extract, (4) Ethanol extract and (5) Control.


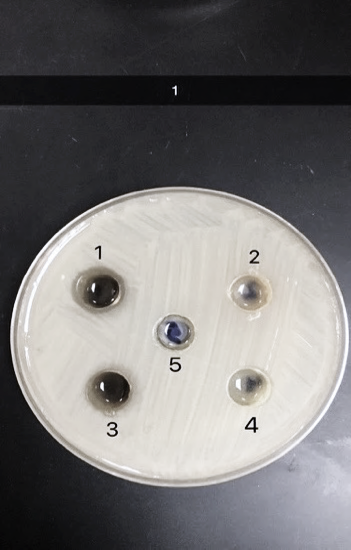


AgNPs

*Cystoseira myrica*

*Staphylococcus aureus*


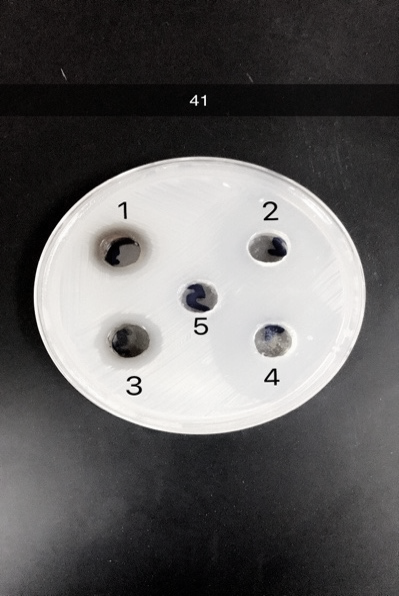


AgNPs

*Ulva rigida*

*Tricosporon cataneum*

Figure (D) Antimicrobial activity of the biosynthesized AgNPs capped by *U. rigida*, against tested pathogen microorganisms *Tricosporon cataneum* indicated by clear zone diameter (CZD, mm). (1) Aqueous extract, (2) AgNPs aqueous, (3) Ethanol extract ,(3) AgNPs Ethanol extract, and (5) Control.


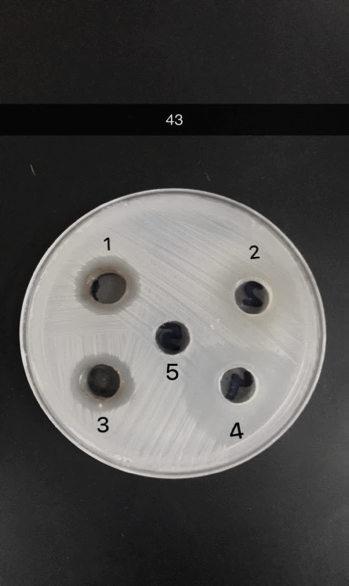


AgNPs

*Cystoseira myrica*

*Tricosporon cataneum*

Figure (E) Antimicrobial activity of the biosynthesized AgNPs capped by *C. myrica*, against tested pathogen microorganisms *Tricosporon cataneum* indicated by clear zone diameter (CZD, mm). (1) AgNPs aqueous, (2) Aqueous extract, (3) Ethanol extract (4) AgNPs Ethanol extract, and (5) Control.


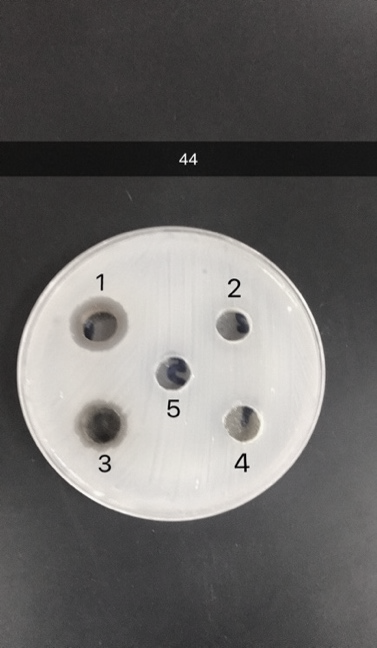


AgNPs

*Gracilaria foliifera*

*Tricosporon cataneum*

Figure (F) Antimicrobial activity of the biosynthesized AgNPs capped by *G. foliifera*, against tested pathogen microorganisms *Tricosporon cataneum* indicated by clear zone diameter (CZD, mm). (1) AuNPs aqueous, (2) Aqueous extract, (3) AuNPs Ethanol extract, (4) Ethanol extract and (5) Control.


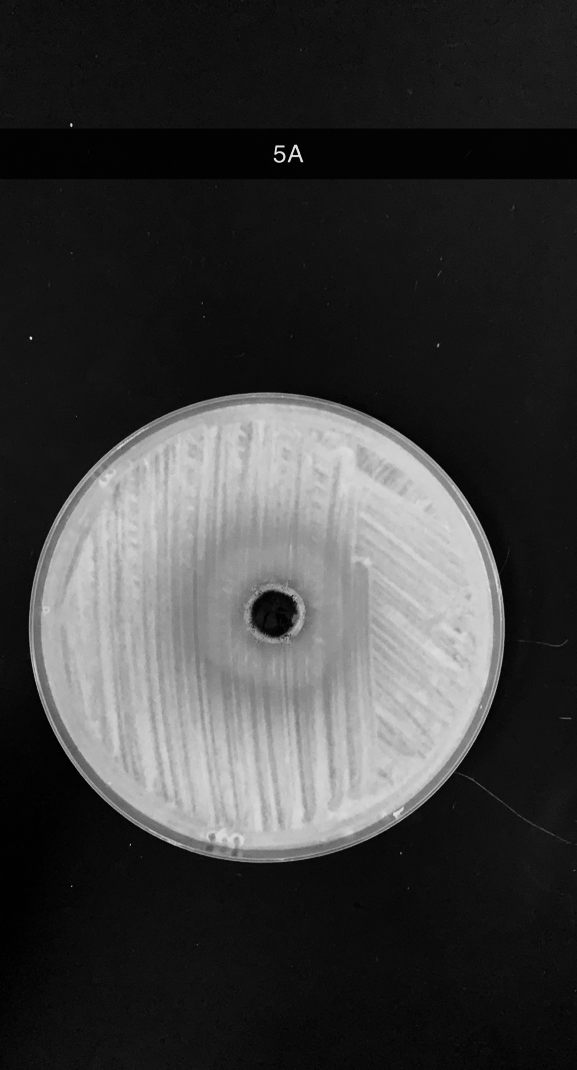


AgNPs

*U. rigida*

*Trichophyton mantigrophytes*

**1**


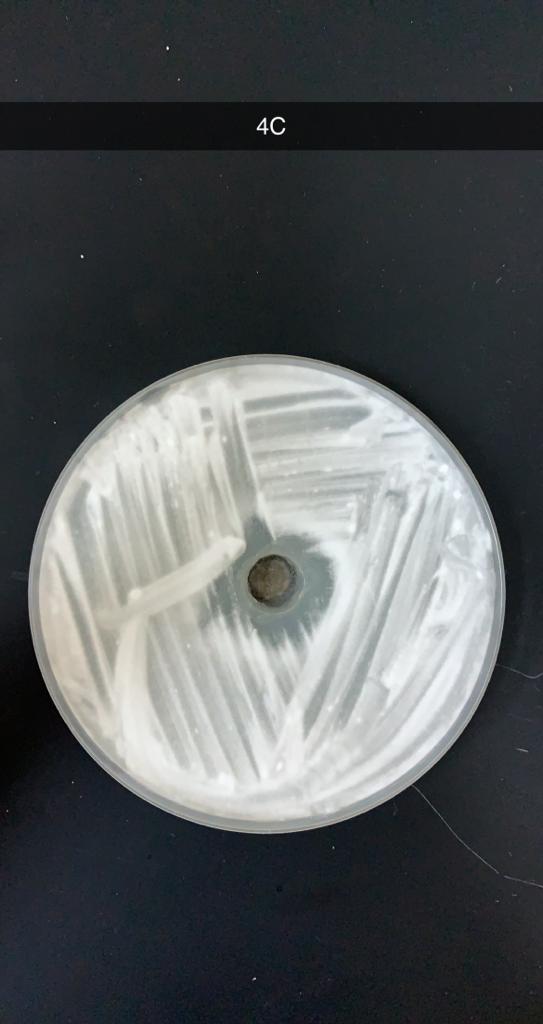


AgNPs

*G. foliifera*

*Trichophyton mantigrophytes*

**2**

Figure (G) Antimicrobial activity by *Trichophyton mantigrophytes* of AgNPs and AuNPs capped by different algal species (1) Chlorophyta (*U. rigida*), (2) Rhodophyta (*G. foliifera*) indicated by clear zone diameter (CZD, mm).
